# Role of Antioxidants in Modulating BDNF Signaling and Oxidative Stress in Glioblastoma: Current Evidence and Therapeutic Perspectives

**DOI:** 10.3390/cells15212006

**Published:** 2026-11-04

**Authors:** Alka Ashok Singh

**Affiliations:** Department of Life Sciences, Yeungnam University, Gyeongsan 38541, Republic of Korea; alkasingh10f@yu.ac.kr

**Keywords:** antioxidants, brain-derived neurotrophic factor, oxidative stress, glioblastoma, polyphenols, neuroprotection, redox modulation, curcumin

## Abstract

**Highlights:**

**What are the main findings?**

- Oxidative stress, Nrf2-mediated redox adaptation, and BDNF/TrkB signaling have independently been implicated in glioma biology, treatment resistance, and neuronal responses; however, direct mechanistic interactions among these pathways, particularly following antioxidant exposure, remain insufficiently characterized in glioblastoma.
- Natural antioxidants, including curcumin, resveratrol, EGCG, hydroxytyrosol, and quercetin, have demonstrated redox-modulating or neurotrophic effects in different experimental systems. Although selected compounds also exhibit antiglioma activities, direct evidence establishing BDNF/TrkB signaling as a mediator of these antitumor effects remains insufficient.

**What are the implications of the main findings?**

- Antioxidant-based strategies may serve as adjuncts to standard glioblastoma therapy, with potential applications in chemosensitization, radiosensitization, neuroprotection, and cognitive preservation.
- Biomarker-guided treatment, improved drug-delivery systems, and well-designed clinical trials are needed to determine appropriate doses, identify responsive patient subgroups, and establish the safety and therapeutic value of these approaches.

**Abstract:**

Glioblastoma (GBM) remains one of the most aggressive and lethal brain tumors, with limited treatment success despite standard multimodal therapy. Oxidative stress and dysregulated brain-derived neurotrophic factor (BDNF) signaling contribute to GBM progression, therapeutic resistance, and treatment-related neurotoxicity. Therefore, this review aims to examine the complex interplay among antioxidants, BDNF/TrkB signaling, and redox imbalance in GBM, focusing on the potential biological activities of natural antioxidants, particularly polyphenols such as curcumin, resveratrol, and epigallocatechin-3-gallate (EGCG). Preclinical studies suggest that these compounds may modulate oxidative stress, influence glioma cell survival, and alter signaling pathways associated with chemoradiotherapy sensitivity and neuronal protection. Nevertheless, direct evidence that BDNF/TrkB signaling mediates the antitumor effects of these antioxidant compounds in glioblastoma remains insufficient, and observations from non-GBM models should be regarded as mechanistic hypotheses rather than validated therapeutic mechanisms. However, clinical evidence supporting their efficacy in patients with GBM remains limited and inconclusive. Human findings from observational cohorts and clinical trials involving non-GBM populations provide indirect insights into antioxidant-related cognitive and redox outcomes but cannot establish therapeutic benefits in glioblastoma. Furthermore, the dual role of BDNF/TrkB signaling in neuronal protection and tumor progression raises concerns regarding generalized BDNF enhancement. Challenges involving bioavailability, optimal dosing, tumor-specific effects, and potential interactions with conventional treatments further limit clinical translation. Future research should prioritize GBM-specific randomized controlled trials, biomarker-guided strategies, and the simultaneous evaluation of neuroprotective and oncological outcomes. Targeting the antioxidant-BDNF-redox axis remains an experimental therapeutic approach that requires rigorous validation before clinical implementation.

## 1. Introduction

Glioblastoma (GBM), is one of the most aggressive primary malignant brain tumors in adults and remains a major challenge in neuro-oncology because of its poor prognosis and limited therapeutic options [1]. Despite maximal multimodal therapy, GBM remains the most common and lethal primary malignant brain tumor in adults, with a median survival time of approximately 15 months. According to WHO CNS5, glioblastoma is classified as an adult-type diffuse glioma, IDH-wildtype, CNS WHO grade 4. Throughout this review, the term glioblastoma (GBM) refers specifically to this entity. Findings obtained from pediatric-type diffuse high-grade gliomas, IDH-mutant astrocytomas, or historical glioblastoma cohorts are considered separately where relevant, because these tumors may represent biologically and diagnostically distinct entities. In adult patients, glioblastoma, IDH-wildtype, may be diagnosed in an appropriate diffuse astrocytic tumor context based on histological evidence of microvascular proliferation or necrosis. In the absence of these histological features, the presence of a TERT promoter mutation, EGFR amplification, or combined whole-chromosome 7 gain and whole-chromosome 10 loss (+7/−10) can establish the diagnosis of glioblastoma, IDH-wildtype, under WHO [2]. Moreover, O6-methylguanine-DNA methyltransferase (MGMT) promoter methylation is a recognized predictive and prognostic biomarker linked to response to alkylating chemotherapy, specifically temozolomide, although it is not a defining diagnostic criterion under WHO CNS5 [3]. Similarly, phosphatase and tensin homolog (PTEN) loss is involved in glioblastoma-associated molecular dysregulation but is not an independent defining diagnostic criterion in the WHO 2021 classification. Importantly, WHO CNS5 separates IDH-mutant astrocytoma, which is categorized as a distinct adult-type diffuse glioma entity, from glioblastoma, IDH-wildtype. As a result, molecular and therapeutic discoveries from IDH-mutant astrocytomas should not be applied straight to glioblastoma without first considering their unique molecular properties [2].

GBMs account for approximately 49% of malignant brain tumors, compared with 30% for lower-grade gliomas. Other malignant central nervous system (CNS) tumors include meningiomas (2%), ependymomas (3%), and primary CNS lymphoma (7%). Common symptoms of malignant brain tumors include headaches (50%), seizures (20–50%), neurocognitive impairment (30–40%), and focal neurologic deficits (10–40%). Magnetic resonance imaging is the preferred method for assessing these tumors with gadolinium-based contrast agents [4]. Despite aggressive standard-of-care treatment, including maximal surgical resection followed by concurrent radiotherapy and temozolomide chemotherapy, the prognosis remains dismal [5]. Patients with GBM continue to have extremely poor survival rates, with 5-year survival rates ranging from 2% to 10% [6]. This poor outcome highlights the urgent need for novel therapeutic strategies that complement existing treatments and improve survival and quality of life.

Oxidative stress is a key contributor to GBM aggressiveness. Reactive oxygen species (ROS) regulate cellular stability by influencing various signaling pathways. Redox systems typically protect cells from oxidative damage; however, gliomagenesis severely impairs cellular redox mechanisms. ROS production in tumor cells influences the cell cycle, contributing to tumor progression and drug resistance in GBM. However, excess ROS production can activate cell death pathways, such as apoptosis and autophagy. Since GBM cells have a high metabolic rate and produce abundant ROS, metabolic adaptation is critical for preventing oxidative stress-induced cell death. The tumor microenvironment, including stromal components, further enhances oxidative stress, promoting tumor progression and drug resistance [7]. Other components of GBM cells include mitochondria, which participate in various cellular processes, particularly resistance to apoptosis, given the prevalence of glycolysis and altered energy metabolism in GBM. Shen et al. report that dichloroacetate restores mitochondrial activity and significantly increases the cytotoxicity of GBM cells when combined with a mitochondrial toxin. Apoptosis mechanisms involve ROS production, as antioxidant treatment reduces the number of apoptotic cells, similar to the effects of glycolysis inhibitors [8].

GBM cells frequently adapt to chronic oxidative environments by upregulating antioxidant pathways, such as Nrf2, allowing them to withstand the treatment-induced adverse conditions. This redox imbalance promotes glioma formation and progression and contributes significantly to treatment failure and recurrence. NRF2 knockdown has been shown to reduce glioblastoma cell proliferation through glutathione depletion and impaired AKT signaling [9]. Separately, NRF2 inhibition has been associated with enhanced temozolomide sensitivity in experimental glioma cells [10]. Separately, NRF2 knockdown increased radiation sensitivity in experimental glioma neurosphere models, particularly following low-dose-rate gamma irradiation [11]. A study shows that NRF2 knockdown in neurosphere cells increases sensitivity to low-dose-rate radiation (3.5 Gy) and high-dose-rate gamma radiation (8 Gy), potentially due to decreased antioxidant response. NRF2 knockdown reduces antioxidant properties of neurosphere cells, resulting in decreased self-renewal and increased differentiation. NRF2 knockdown significantly increases neurosphere sensitivity to low-dose-rate irradiation and, partly, high-dose-rate gamma irradiation (only at the highest dose of 8Gy). Neurospheres exhibit increased sensitivity to low-dose-rate gamma irradiation, inducing oxidative stress [11,12].

Similarly, brain-derived neurotrophic factor (BDNF), its high-affinity receptor tropomyosin receptor kinase B (TrkB), and the p75 neurotrophin receptor mediate various biological functions in the healthy and pathological brain. Over the past two decades, BDNF and its receptor TrkB have been overexpressed in various tumors [13,14]. In the normal CNS, BDNF/TrkB signaling is required for neuronal survival, synaptic plasticity, differentiation, and cognitive function. It activates downstream pathways, such as PI3K/Akt, MAPK/ERK, and PLCγ, promoting cell survival and neuroprotection [15]. Studies demonstrate high BDNF expression in the hypothalamus, cortex, hippocampus, amygdala, basal forebrain, dorsal vagal complex, and ventral tegmental area [16]. BDNF is highly expressed and concentrated in the CNS and plasma [17]. However, the source of peripheral and circulating BDNF remains unknown. In cancer, BDNF/TrkB signaling frequently acquires oncogenic properties [18]. The invasive GBM cell subpopulation exhibits migration patterns similar to those of immature neurons. Genuine synapses form between presynaptic neurons and postsynaptic glioma cells through electrical and chemical signaling, promoting tumor growth and invasion [19,20,21]. These synapses connect neurons to glioma cells through AMPA receptor-mediated excitatory postsynaptic currents. AMPA receptor inhibitors effectively reduce glioma cell proliferation and invasion [20]. Another type of synapse, similar to the astrocyte-mediated tripartite synapses, is involved in breast-to-brain metastasis [22]. Glutamate signaling is essential for both types of synapses. In the GBM microenvironment, aberrant production of glutamate, a paracrine signaling factor, may contribute to neuronal hyperexcitability and tumor growth in GABAergic interneurons [23,24]. Through the BDNF–TrkB pathway, paracrine BDNF secretion regulates the number of neuron-to-glioma synapses and AMPA receptor trafficking to the glioma cell membrane, increasing the amplitude of glutamate-evoked currents. Neuron-to-glioma synaptic connections are reduced, neuronal activity-induced glioma proliferation is inhibited, and survival is prolonged when BDNF secretion is blocked, or glioma TrkB expression is inhibited [25].

The dynamic interplay between oxidative stress and antioxidant mechanisms is critical for understanding GBM biology and developing new therapeutic approaches. The interplay among oxidative stress, antioxidant systems, and BDNF/TrkB signaling in GBM suggests new avenues for intervention. Natural polyphenols and other redox-modulating compounds have been reported to influence oxidative stress and neurotrophic signaling in different experimental models. However, evidence demonstrating that antioxidant-induced changes in BDNF/TrkB signaling directly mediate glioblastoma progression or treatment responses remain insufficient. Therefore, antioxidant-associated neurotrophic effects observed in non-neoplastic experimental systems should be distinguished from experimentally validated antiglioma mechanisms [26]. Understanding these connections is essential for developing safer and more effective supportive therapies that may preserve healthy brain tissue while making tumor cells more susceptible to standard treatments. Polyphenols are a class of naturally occurring phytochemicals found in high concentrations in fruits, vegetables, and other natural products and characterized by multiple hydroxyl groups on aromatic rings. These compounds are classified into two types—flavonoids and nonflavonoids—based on the number of phenol rings and their interactions [26]. In glioblastoma research, oxidative stress, antioxidant defense mechanisms, and BDNF/TrkB Signaling have all garnered a lot of attention on their own, but their possible interconnections within the framework of antioxidant-based therapies are still not fully explored. Previous research has identified the role of redox imbalance and Nrf2-dependent antioxidant adaptation in glioblastoma progression and treatment resistance, while separate studies have shown the significance of BDNF/TrkB Signaling in neuronal survival, synaptic plasticity, and glioma-associated proliferative Signaling.

The present study stands out for its comprehensive and critical assessment of the links between antioxidant-mediated redox regulation, Nrf2-dependent cellular adaptability, and BDNF/TrkB Signaling in glioblastoma [7]. However, it is unclear whether antioxidant-induced BDNF expression modifications directly cause anticancer effects in glioblastoma. Thus, this review focuses on three central research questions: (i) How do oxidative stress, Nrf2-mediated antioxidant responses, and BDNF/TrkB signaling interact in glioblastoma, and which of these interactions have been experimentally validated? (ii) Under what cellular and therapeutic settings may antioxidant-associated BDNF/TrkB signaling regulation promote glioma cell survival, malignant progression, or therapeutic resistance while simultaneously supporting neuronal protection? (iii) Rather than drawing conclusions from neurological or other non-GBM models, to what degree are the suggested effects of natural antioxidants on BDNF-dependent chemosensitization, radiosensitization, and neuroprotection supported by direct GBM-specific evidence? By addressing these issues, the current review aims to identify evidence gaps that necessitate additional mechanistic and clinical research, critically assess the possible therapeutic risks of generalized BDNF enhancement, and differentiate BDNF-associated molecular changes from experimentally demonstrated BDNF-dependent mechanisms.

Therefore, this review critically examines the proposed antioxidant-BDNF-redox axis in glioblastoma, emphasizing the mechanistic relationships among oxidative stress, Nrf2 signaling, and BDNF/TrkB activity; the opposing implications of neuronal protection and tumor-promoting responses; and the distinction between experimentally validated findings and indirect evidence supporting potential therapeutic applications.

## 2. Literature Search Strategy and Review Methodology

This article was developed as a narrative review to critically examine the interactions among oxidative stress, antioxidant defense mechanisms, brain-derived neurotrophic factor (BDNF)/tropomyosin receptor kinase B (TrkB) signaling, and glioblastoma (GBM) progression. The molecular mechanisms driving the context-dependent effects of natural antioxidants were studied in depth, including their potential neuroprotective properties and contributions to tumor adaptability and therapeutic resistance. Relevant publications were identified through literature searches conducted in PubMed, Scopus, Web of Science, and Google Scholar, covering the period from 2000 to 30 September 2026. The search strategy included combinations of the following terms: “glioblastoma,” “glioma,” “oxidative stress,” “reactive oxygen species,” “Nrf2,” “Keap1,” “BDNF,” “TrkB,” “antioxidants,” “polyphenols,” “curcumin,” “resveratrol,” “epigallocatechin gallate,” “temozolomide,” “radiotherapy,” “neuroprotection,” and “therapeutic resistance.” Relevant studies focused on oxidative stress regulation, Nrf2-mediated antioxidant defense, BDNF/TrkB signaling, or the biological effects of natural antioxidants in glioblastoma and comparable experimental models. Priority was given to original scientific publications presenting mechanistic investigations, glioma cell-line experiments, patient-derived models, animal studies, and clinical observations involving glioblastoma or GBM patients. Review articles were primarily utilized to provide context and seek out relevant primary papers. Evidence from research on other cancers, neurological illnesses, or general population cohorts was evaluated only if it provided important mechanistic or contextual data. Such findings were clearly differentiated from GBM-specific evidence in order to avoid improper extrapolation of therapy effects. Each study’s experimental paradigm, intervention characteristics, measurable endpoints, and limitations were all carefully considered. Given the narrative design of this review, no quantitative meta-analysis was performed. The review does not establish the clinical efficacy of antioxidant-based interventions in GBM; rather, it critically summarizes the available evidence and identifies mechanistic uncertainties, translational limitations, and priorities for future investigation.

## 3. Oxidative Stress and Redox Imbalance in Glioblastoma

GBM cells are exposed to persistent oxidative stress, characterized by increased ROS production exceeding the capacity of cellular antioxidant defenses. These cells also exhibit elevated basal ROS levels and oxidative stress, indicating an imbalance between ROS generation and antioxidant defenses that promotes tumor cell proliferation, migration, and therapeutic resistance [27]. This redox imbalance defines GBM biology and contributes to its aggressive phenotype [28]. In contrast to normal cells, which tightly regulate ROS levels, GBM cells experience chronic oxidative stress caused by rapid proliferation, metabolic alterations, hypoxia, and mitochondrial dysfunction [29].

In GBM, elevated ROS levels actively support several important cancer hallmarks [30]. Moderate-to-high ROS levels promote tumor cell proliferation by activating mitogenic signaling pathways, such as PI3K/Akt and MAPK/ERK [31]. They also promote invasion and migration by oxidizing proteins involved in cytoskeletal remodeling and upregulating matrix metalloproteinases (MMPs) [32,33,34]. ROS promotes angiogenesis by stabilizing hypoxia-inducible factor-1α (HIF-1α) and increasing VEGF expression, resulting in abnormal, leaky blood vessels characteristic of GBM [35,36,37]. Furthermore, ROS promotes the release of immunosuppressive cytokines, polarize macrophages and microglia toward a pro-tumorigenic M2 phenotype, and suppress antitumor immune responses, thereby facilitating immune evasion [38,39,40].

Excessive oxidative stress causes cell death, despite ROS also supporting tumor growth [41]. GBM cells have evolved adaptive mechanisms to retain redox homeostasis at a level that promotes survival and therapeutic resistance [7]. This “redox paradox” enables tumor cells to benefit from oxidative stress while avoiding cytotoxicity [42,43].

### 3.1. Antioxidant Defense Systems and Redox Adaptation in Glioblastoma

The antioxidant systems in GBM are markedly dysregulated [44]. One key component is the Nuclear factor erythroid 2-related factor 2 (Nrf2) pathway [45]. The Nrf2/Kelch-like ECH-associated protein 1 (Keap1) pathway is an important regulator of antioxidant defense in GBM. Under oxidative stress, Nrf2 dissociates from Keap1, translocates to the nucleus, and activates the expression of antioxidant and cytoprotective genes [45]. Nrf2 is frequently overexpressed in GBM, particularly in the mesenchymal subtype and glioma stem cells (GSCs), contributing to poor patient outcomes and therapeutic resistance [46]. Nrf2 upregulation increases the expression of genes involved in glutathione (GSH) synthesis, heme oxygenase-1 (HO-1), and NAD(P)H quinone dehydrogenase 1 (NQO1) [47]. Other important antioxidant enzymes are also altered. Superoxide dismutases (SODs), particularly mitochondrial SOD2, are frequently overexpressed in GBM as a protective mechanism against superoxide radicals [48]. However, this may lead to increased hydrogen peroxide levels, inducing protumor signaling [49]. The GSH system, including reduced GSH, GSH peroxidase (GPx), and GSH reductase, is a key cellular antioxidant buffering mechanism [29]. In GBM, increased GSH levels and GSH metabolism help to maintain redox homeostasis, allowing tumor cells to counteract ROS produced by temozolomide and other therapies, thereby promoting treatment resistance and tumor persistence [50,51]. These dysregulated antioxidant systems enable GBM cells to tolerate high ROS levels while using them as signaling molecules, providing a selective advantage that promotes tumor progression and therapeutic resistance.

Oxidative stress and redox imbalance are key features of GBM malignancy rather than byproducts of its aggressive metabolism. Targeting redox biology may have therapeutic potential because GBM cells use ROS to support proliferation, invasion, angiogenesis, and immune evasion while maintaining viability through enhanced antioxidant defenses, particularly Nrf2, SOD, and GSH pathways. Modulating this redox balance, either by inhibiting key antioxidant responses or increasing oxidative burden, may improve treatment outcomes.

### 3.2. Nrf2 Signaling in Glioblastoma: Regulation of Redox Homeostasis and Therapy Resistance

**Figure 1:**
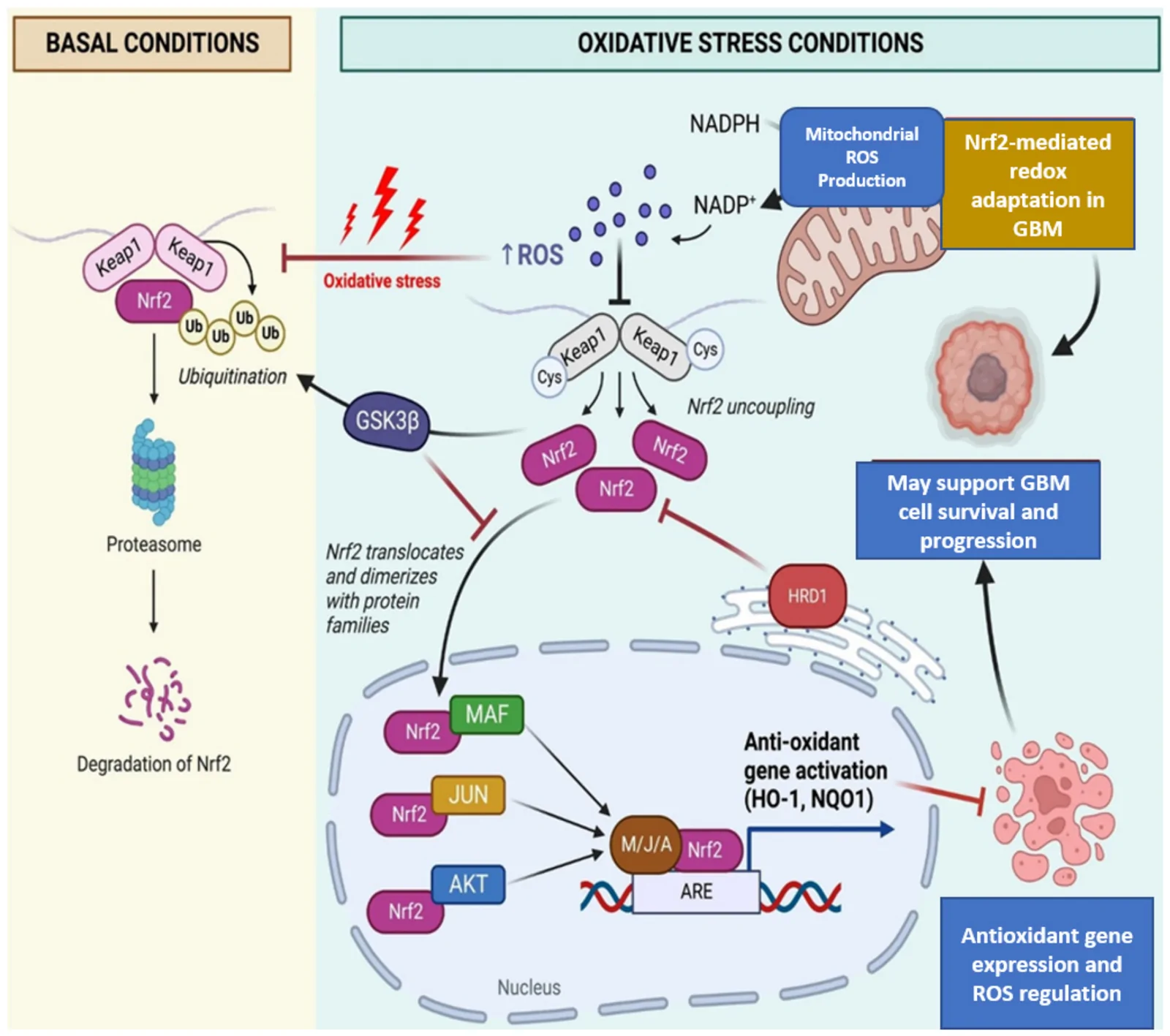
General overview of Nrf2 signaling under basal conditions and oxidative stress and its role in GBM pathogenesis. Nrf2-mediated antioxidant signaling and redox adaptation in glioblastoma. Under basal conditions, Keap1 promotes Nrf2 ubiquitination and proteasomal degradation. Under oxidative stress, increased reactive oxygen species (ROS), including mitochondrial ROS, promote Nrf2 stabilization and nuclear translocation, leading to antioxidant response element (ARE)-dependent expression of cytoprotective genes, including HO-1 and NQO1. These responses regulate cellular redox balance and may support glioblastoma (GBM) cell survival and progression in specific contexts. The mitochondrial NADPH/NADP^+^ pathway illustrates general redox regulation rather than IDH mutation-dependent signaling. Antioxidant gene activation does not necessarily induce apoptosis or directly suppress tumor cells, and the effects of Nrf2 activation remain context-dependent. The schematic focuses on redox regulation relevant to IDH-wildtype GBM. Adapted and modified from Awuah et al. (2022) [56]. Abbreviation: GBM-glioblastoma.

Studies show that Nrf2 and its downstream antioxidant pathways are upregulated in GBM after radiation therapy, and Nrf2 knockdown increases radiation-induced ROS accumulation and apoptosis. These findings suggest that Nrf2 serves as a master regulator of cellular antioxidant defenses and a crucial mediator of redox adaptation in GBM [7]. In normal cells, Nrf2 is tightly controlled by its cytoplasmic inhibitor, Keap1, promoting Nrf2 ubiquitination and proteasomal degradation [45,52,53]. During oxidative stress, ROS alters Keap1, enabling Nrf2 to stabilize, translocate to the nucleus, and bind to antioxidant response elements (AREs) in target gene promoter regions [53,54]. These regulatory mechanisms and their potential implications for Nrf2-mediated redox adaptation in glioblastoma are illustrated in Figure 1.

Nrf2, a transcription factor with a specific role in cancer therapy, is involved in multiple mechanistic cascades that produce antimetabolic, antioxidative, anti-inflammatory, and anticancer effects [55]. However, in GBM, the Nrf2 pathway is frequently constitutively activated, transforming its protective role into a protumorigenic one [56]. Nrf2 is a crucial modulator of cellular defense against DNA damage, autophagy, antioxidant defense, and cell quiescence. Keap1—an adaptor of the E3 ubiquitin ligase complex—binds to Nrf2 under normal circumstances, promoting its ubiquitination and degradation. ROS oxidize important cysteine residues in Keap1 during oxidative stress, interfering with its ability to interact with Nrf2. After stabilization, Nrf2 translocates to the nucleus, dimerizes with Maf proteins, and binds to AREs to activate genes related to metabolism, detoxification, and antioxidant defense. Consequently, mutations affecting Keap1 or Nrf2 disrupt redox homeostasis and promote tumor growth [57]. Nrf2 activates the transcription of numerous antioxidant and detoxifying genes, including glutamate-cysteine ligase catalytic and modifier subunits involved in GSH synthesis, HO-1, NQO1, SODs, and GPx [58]. The resulting increase in GSH levels and antioxidant capacity enables tumor cells to maintain redox homeostasis at a level favorable for survival and proliferation.

Beyond antioxidant defense, Nrf2 promotes several cancer-related characteristics in GBM cells, stimulating cell proliferation and survival through the PI3K/Akt and MAPK/ERK signaling pathways [59]. Nrf2 also promotes tumor invasion and migration by activating MMP-2 and MMP-9 and facilitating epithelial-to-mesenchymal transition [60,61]. In hypoxic GBM regions, Nrf2 and HIF-1α promote angiogenesis by increasing VEGF expression [56,62]. Furthermore, Nrf2 promotes immune evasion by polarizing tumor-associated microglia and macrophages toward an immunosuppressive M2 phenotype and promoting the survival of GSCs, which are highly resistant to therapy [63,64]. Overall, although the Nrf2 pathway is essential for neuroprotection in healthy brain tissue, aberrant hyperactivation in GBM promotes tumor growth, redox adaptation, and treatment resistance. This duality makes Nrf2 a significant but challenging therapeutic target. Strategic modulation of Nrf2 activity represents a promising redox-based approach to GBM treatment, involving selective inhibition in tumor cells or controlled activation for neuroprotection.

Experimental data supports Nrf2’s role in glioblastoma cell adaptability, stem-like cell maintenance, and treatment resistance. The study found that Nrf2 is essential for the self-renewal capacity of glioma stem cells, implying that prolonged Nrf2 signaling may contribute to the persistence of tumor cell populations associated with recurrence and resistance to therapy [65]. Similarly, another study found that inhibiting Nrf2 improved the anticancer effects of temozolomide (TMZ) in glioma cells, possibly through modulation of Ras/Raf/MEK signaling [10]. These findings suggest that Nrf2-mediated antioxidant defense can provide a survival advantage to malignant cells under treatment stress, underlining the potential limitations of therapies targeted at increasing overall antioxidant activity in glioblastoma.

However, the effects of Nrf2 activation are not universally tumor-protective and may be dependent on the mechanisms of cell death generated by various therapeutic approaches. de Souza et al. found that enhanced Nrf2 expression in TMZ-resistant T98G glioma cells increased their vulnerability to ferroptosis under certain experimental conditions. This effect was associated with upregulation of the ATP-binding cassette transporter ABCC1/MRP1 and increased glutathione depletion following inhibition of the system xc− cystine transport pathway. Interestingly, inhibiting Nrf2 increased TMZ sensitivity while decreasing vulnerability to ferroptosis in the same experimental setting. These findings suggest that Nrf2-dependent metabolic adaptation can both increase resistance to conventional chemotherapy and generate vulnerabilities to alternate cell-death pathways [66]. These context-dependent effects highlight the underlying antioxidant challenge in glioblastoma. Although Nrf2 activation promotes cellular protection against oxidative damage and may be advantageous in the preservation of nonmalignant brain tissue, prolonged activation of the same cytoprotective pathways in glioblastoma cells may aid tumor survival and treatment resistance. This contradiction is especially pertinent for natural polyphenols like curcumin, resveratrol, and epigallocatechin-3-gallate, which might alter Nrf2-associated redox signaling. Their biological effects can differ depending on the cellular environment, concentration, treatment duration, and interactions with other signaling pathways. As a result, the antioxidant characteristics of these drugs should not be construed as intrinsically advantageous in glioblastoma, and Nrf2 activation should not be used as evidence of therapeutic success.

### 3.3. Kelch-like ECH-Associated Protein 1Mutations and Their Role in Glioblastoma

KEAP1 is the primary negative regulator of Nrf2 [67,68]. Under normal conditions, KEAP1 binds cytoplasmic Nrf2 and facilitates its ubiquitination and subsequent proteasomal degradation [69,70]. Loss-of-function mutations in KEAP1 stabilize and constitutively activate Nrf2, resulting in persistent upregulation of antioxidant and cytoprotective genes [71].

In GBM, genetic alterations in the KEAP1-NRF2 pathway are rare, with mutations in KEAP1 or NFE2L2 detected in only 11 of 721 TCGA brain tumors (~1.5%). This finding indicates that constitutive Nrf2 activation in GBM primarily arises from nonmutational mechanisms rather than direct genetic alterations [72]. Promoter hypermethylation and loss of heterozygosity may contribute to KEAP1 inactivation in certain tumors. Nrf2 hyperactivation is observed in some mesenchymal GBM [73,74,75]. KEAP1 dysfunction constitutively activates Nrf2, providing GBM cells with multiple survival advantages [76]. It increases the expression of key antioxidant genes, including GCLC, GCLM, HO-1, NQO1, and glutathione-related enzymes, thereby enabling tumor cells to maintain redox homeostasis despite elevated ROS caused by rapid proliferation, hypoxia, and metabolic stress [77,78]. This redox adaptation supports key cancer hallmarks, including sustained proliferation, invasion, angiogenesis, and immune evasion [79]. Furthermore, KEAP1-deficient tumors show greater resistance to standard therapies, particularly TMZ and ionizing radiation, by reducing oxidative damage-induced apoptosis and increasing DNA repair [80]. KEAP1 mutations are associated with GSC persistence, potentially contributing to treatment resistance and tumor recurrence [81]. Interactions between KEAP1/Nrf2 signaling and other pathways, including HIF-1α under hypoxia and p62/SQSTM1-mediated positive feedback, further promote the aggressive GBM phenotype [82,83]. Overall, functional KEAP1 inactivation leads to Nrf2 hyperactivation, supporting redox adaptation, therapy resistance, and tumor aggressiveness despite the rarity of direct KEAP1 mutations in GBM. Therefore, KEAP1 status should be assessed for guiding personalized antioxidant-based or Nrf2-targeted therapeutic strategies for GBM.

## 4. Brain-Derived Neurotrophic Factor in Brain Cancer: Friend or Foe?

BDNF and its primary receptor, TrkB, are important for normal neural development and plasticity, while increasing evidence suggests that aberrant BDNF/TrkB signaling promotes glioma progression and tumor cell survival. In gliomas, BDNF-induced TrkB activation enhances brain tumor stem cell viability through ERK and Akt signaling, highlighting the BDNF/TrkB axis as a potential therapeutic target [84].

### 4.1. BDNF as a Potential Biomarker

BDNF and TrkB expression have been investigated as potential tissue-associated biomarkers in glioma, although their diagnostic, prognostic, and treatment-predictive significance remains incompletely validated. Associations between mature BDNF expression in resected glioma tissue and tumor grade should be distinguished from measurements of circulating BDNF in serum or plasma, which may reflect different biological sources and are influenced by methodological and physiological factors [85,86]. Mature BDNF expression is higher in high-grade gliomas, including GBM, than in low-grade gliomas and normal brain tissue. Increased mature BDNF expression is also positively associated with glioma malignancy grade, suggesting that the BDNF/TrkB axis may contribute to glioma progression. Study shows that TrkB and mature BDNF are correlated. High-grade gliomas exhibited markedly elevated mature BDNF expression, which correlated positively with tumor malignancy and TrkB receptor expression [87]. These findings support further investigation of tissue BDNF/TrkB expression as a potential marker associated with glioma grade; however, its independent prognostic value and clinical utility remain insufficiently established. However, the potential of BDNF as a noninvasive biomarker for prognosis, tumor grading, and monitoring treatment response or recurrence remains uncharacterized. Before BDNF can be routinely utilized in clinical practice, larger, standardized clinical validation studies are required due to inconsistent findings across studies. The potential clinical applications of BDNF should be considered separately. A diagnostic biomarker must be able to distinguish patients with glioblastoma from appropriate controls, whereas a prognostic biomarker must have a correlation with clinical outcomes irrespective of known prognostic markers. In contrast, a treatment-predictive biomarker must show a link with differences in responses to a certain therapeutic intervention. Although BDNF expression has been linked to glioma grade and tissue-associated molecular alterations, its diagnostic, prognostic, and treatment-predictive usefulness in glioblastoma is not fully confirmed [85,88]. Furthermore, measurements of BDNF in tumor tissue, serum, and plasma should not be employed interchangeably. Circulating BDNF concentrations may be influenced by platelet storage and release, serum clotting conditions, anticoagulant selection, centrifugation procedures, and assay variability [85]. Patient-specific characteristics, such as age and gender, may potentially contribute to variations in circulating BDNF values [85]. As a result, cross-study comparisons necessitate standardized sampling and analytical methods, proper controls, and careful evaluation of any confounding factors. Until prospective clinical validation is available, evaluating BDNF/TrkB expression to select patients for antioxidant-based or other targeted therapies should be considered exploratory research rather than an established clinical strategy.

### 4.2. Pro-Tumorigenic Effects: Promotion of Glioma Cell Survival, Migration, and Therapy Resistance

In the tumor context, BDNF/TrkB signaling frequently has pro-tumor effects. Mature BDNF increases glioma cell survival by activating key pro-survival pathways, including PI3K/Akt and MAPK/ERK, thereby suppressing apoptosis [89]. The research about BDNF-mediated PI3K/AKT pathway in glioblastoma shows In GBM tissues and cells, BDNF was upregulated while MiR-489-3p was downregulated. GBM cell invasion, migration, and proliferation were all decreased by MiR-489-3p re-expression or BDNF knockdown, whereas apoptosis was encouraged. MiR-489-3p targeted BDNF, and BDNF up-regulation counteracted the effects of miR-489-3p on GBM cells. Overexpression of miR-489-3p significantly decreased the protein levels of p-AKT and p-PI3K in GBM cells, whereas BDNF up-regulation restored these levels. Therefore, by inhibiting the BDNF-mediated PI3K/AKT pathway in GBM, miR-489-3p suppressed proliferation, migration, and invasion and triggered apoptosis, offering novel approaches for GBM therapeutic treatment [90]. Paracrine signaling is an important mechanism, with active neuron-induced BDNF binding to TrkB receptors on GSCs, activating downstream MAPK/ERK and PI3K/AKT signaling and promoting tumor cell survival and plasticity. These interactions may sustain stem-like tumor populations and stimulate tumor regrowth. Furthermore, BDNF/TrkB signaling may contribute to therapy resistance by increasing tumor cell survival and decreasing the cytotoxic effects of chemoradiation. These pro-tumorigenic effects suggest that the BDNF/TrkB axis may be a therapeutic target for inhibiting glioma progression and enhancing treatment outcomes [91].

### 4.3. Neuroprotective Effects: Potential to Reduce Treatment-Induced Cognitive Impairment

Conversely, BDNF has neuroprotective effects in the brain tumor setting [92]. Standard therapies, such as cranial radiation and chemotherapy, frequently cause substantial cognitive impairment, including memory loss and reduced executive function [93]. BDNF promotes neuronal survival, synaptic plasticity, and neurogenesis, potentially alleviating these adverse effects [92]. Preclinical data suggest that increasing BDNF levels may protect normal brain tissue from treatment-induced neurotoxicity and improve cognitive outcomes in cancer survivors [94]. Although BDNF-associated neuroprotective mechanisms are biologically plausible, their ability to preserve cognitive function without simultaneously promoting tumor growth has not been adequately demonstrated in the same glioblastoma-bearing experimental models. Accordingly, the potential safety and therapeutic selectivity of BDNF enhancement remain unresolved.

### 4.4. Interaction with Oxidative Stress

BDNF/TrkB signaling and oxidative stress are independently implicated in glioma biology; however, direct experimental evidence establishing bidirectional mechanistic regulation between these pathways in glioblastoma remains limited. BDNF-associated antioxidant and neuroprotective responses have been reported in non-neoplastic neural experimental systems, but these observations do not establish that BDNF/TrkB signaling directly regulates redox adaptation or treatment resistance in glioblastoma cells [95]. Malignant glioma cell survival is facilitated by BDNF/TrkB signaling, which activates downstream ERK and Akt pathways. TrkB suppression decreased neurotrophin-dependent growth in brain tumor-initiating cells originating from human malignant gliomas, but BDNF activation of TrkB increased cell viability. This suggests that BDNF/TrkB signaling has a direct pro-survival role in gliomas [96]. GBM cells independently display redox-adaptive pathways that support survival in hypoxic and treatment-induced oxidative stress. Through PTEN oxidation and downstream Akt activation, NADPH oxidase-derived ROS have been demonstrated to promote GBM development and radiation resistance, whereas NOX signaling suppression decreased tumor growth and improved radio sensitivity [97]. Nevertheless, there is currently limited direct proof that BDNF/TrkB itself causes oxidative-stress adaptation in GBM. Therefore, even though redox-dependent treatment resistance and BDNF/TrkB-mediated survival signaling are both well-established aspects of GBM biology, more research is needed to determine how they directly intersect mechanistically. Understanding these dynamics is essential for developing antioxidant-based strategies to selectively modulate BDNF signaling in tumors and healthy tissue.

**Figure 2:**
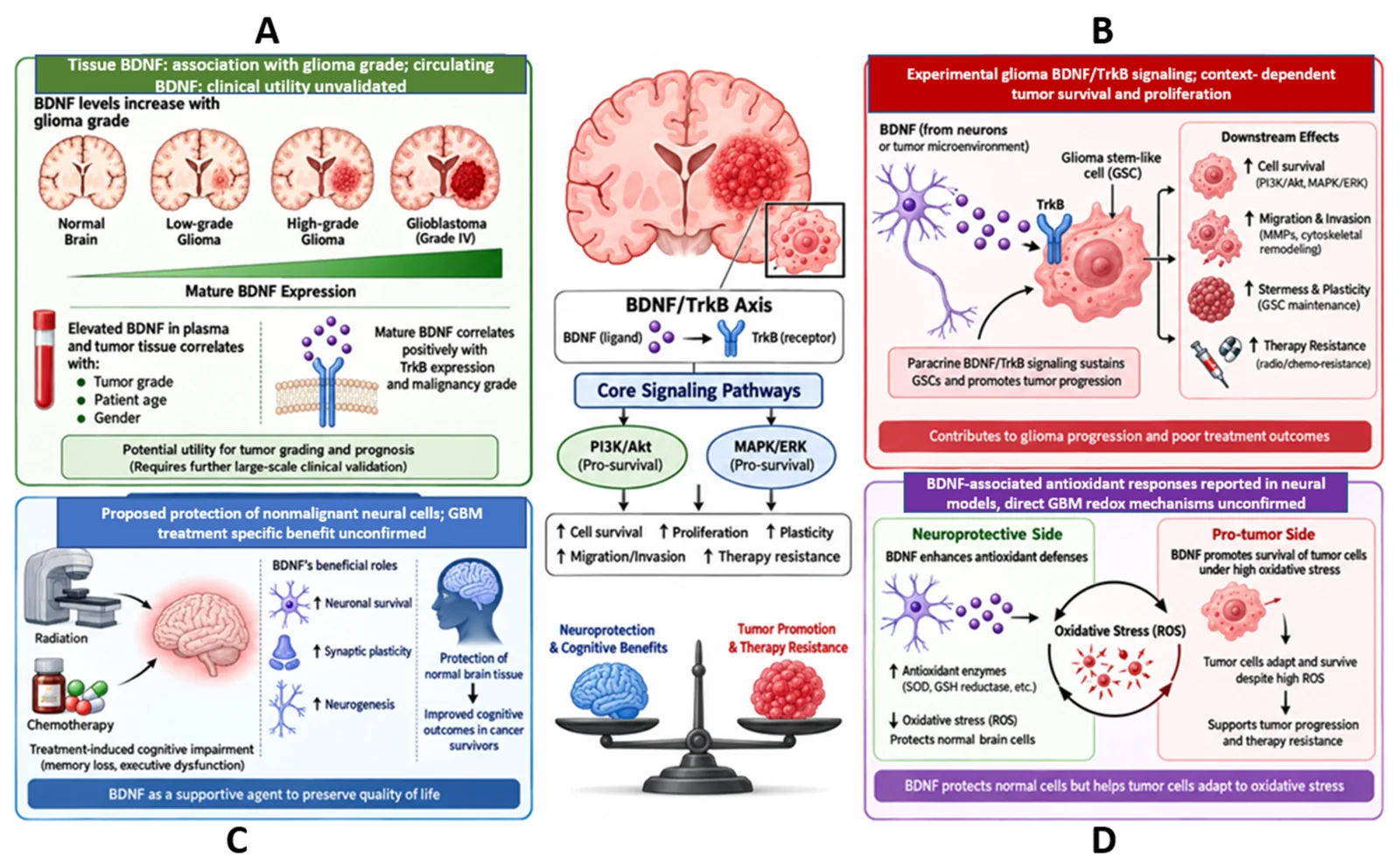
Context-dependent roles of BDNF/TrkB signaling in glioma progression, neuroprotection, and oxidative stress. (**A**) Tissue-associated mature BDNF expression has been correlated with glioma grade in selected studies, whereas circulating BDNF concentrations are influenced by physiological and preanalytical factors. Their clinical diagnostic and prognostic utility in glioblastoma (GBM) remain unvalidated. (**B**) Experimental glioma studies suggest that BDNF/TrkB signaling activates PI3K/Akt and MAPK/ERK pathways, contributing to tumor cell survival, proliferation, migration, invasion, and stem-like properties. Its involvement in therapeutic resistance remains context-dependent. (**C**) BDNF supports neuronal survival, synaptic plasticity, and neurogenesis in non-neoplastic neural systems; however, its potential to prevent treatment-related cognitive impairment without promoting GBM progression remains unconfirmed. (**D**) BDNF-associated antioxidant responses have been reported in non-neoplastic neurological models, but direct evidence establishing BDNF/TrkB-mediated redox adaptation and therapeutic resistance in GBM remains insufficient. The schematic illustrates experimentally supported glioma-associated mechanisms alongside proposed neuroprotective and redox-related effects that require further validation. Abbreviations: BDNF-brain-derived neurotrophic factor; TrkB—tropomyosin receptor kinase B; ROS—reactive oxygen species.

Finally, BDNF has a dual role in brain cancer. Its pro-tumor actions promote aggression and therapy resistance, whereas its neuroprotective properties may help minimize treatment-related cognitive impairment. This duality emphasizes the importance of context-specific modulation of BDNF/TrkB signaling and its interaction with redox balance when designing safer and more effective glioma treatments. The context-dependent roles of BDNF/TrkB signaling in glioma-associated biomarker research, tumor progression, proposed neuroprotection, and oxidative stress regulation are summarized in Figure 2.

### 4.5. The Therapeutic Paradox of BDNF Enhancement in Glioblastoma: Balancing Neuroprotection and Tumor Progression

In glioblastoma (GBM), brain-derived neurotrophic factor (BDNF) signaling poses a basic therapeutic conundrum because the same molecular pathways that support neuronal survival, synaptic plasticity, and cognitive function may also be involved in the tumor’s growth. When BDNF binds to its high-affinity receptor, tropomyosin receptor kinase B (TrkB), it triggers downstream signaling cascades that promote neuronal survival and adaptive plasticity. These cascades include PI3K/Akt, MAPK/ERK, and PLCγ. BDNF’s neuroprotective potential has drawn special interest in relation to cognitive impairment brought on by cancer treatment. A systematic study by Ng et al. showed relationships between BDNF-related biomarkers and cognitive outcomes among cancer patients and survivors, however the findings were diverse [98]. Furthermore, El-Khatib et al. found that riluzole-associated BDNF augmentation improved cognitive function, conserved synaptic integrity, and reduced neuroinflammation in a mouse model of cranial irradiation [99]. These findings indicate that BDNF-related therapies may have neuroprotective effects against treatment-induced neurological impairment.

However, because BDNF’s downstream survival pathways may also promote the proliferation and adaptability of malignant cells, its therapeutic stimulus may be a challenge in GBM. The research highlighted the significance of neuron-glioma interactions in tumor progression by showing that glioma cells form functional synaptic connections with neurons and that neuronal electrical activity promotes tumor proliferation. More specifically, Taylor et al. showed that BDNF-TrkB signaling promotes malignant synaptic plasticity and controls neuron-to-glioma synaptic connection. Significantly, TrkB blockage extended longevity in glioma-bearing mice, whereas suppression of activity-regulated BDNF production or inhibition of glioma TrkB signaling decreased tumor progression in experimental models. These findings offer mechanistic proof that increasing BDNF availability in the tumor microenvironment may unintentionally promote carcinogenic signaling and undermine the intended advantages of neuroprotective treatments.

This paradox is especially pertinent to therapeutic approaches relying on antioxidants. The ability of natural polyphenols, such as curcumin, resveratrol, and epigallocatechin-3-gallate, to control oxidative stress, neurotrophic signaling, and cellular survival pathways has been studied. To resolve this therapeutic paradox, it is necessary to distinguish between healthy BDNF signaling in nonmalignant neuronal populations and oncogenic TrkB signaling in glioma cells. Taylor et al.’s experimental findings suggest that tumor-selective TrkB inhibition, along with preservation of neuronal neurotrophic function, could be a promising treatment approach. However, obtaining such selectivity may be difficult since neuronal and malignant cells share multiple signaling pathways, and GBM frequently infiltrates healthy brain tissue. Spatially restricted drug delivery, receptor-specific regulation, and molecular characterization of TrkB-responsive tumor populations may provide future prospects, however their efficacy and safety have yet to be determined. The biological implications of BDNF signaling in glioma are determined by the ligand’s cellular source, responding cell population, BDNF molecular form, and neurotrophin receptor expression profile. BDNF is derived from neurons, tumor cells, or other components of the tumor microenvironment, and its effects vary depending on whether signaling happens in nonmalignant neurons, glioma cells, or cells in the surrounding milieu. In nonmalignant neurons, mature BDNF signaling via full-length TrkB can activate the PI3K/Akt and MAPK/ERK pathways, which are involved with neuronal survival and synaptic plasticity. However, glioma cells that express functioning TrkB receptors may benefit from similar signaling pathways for survival, proliferation, and malignant synaptic plasticity. An additional distinction concerns mature BDNF and its precursor, proBDNF, which can exhibit different receptor preferences and biological effects. While proBDNF can communicate through p75NTR and sortilin-containing receptor complexes, creating context-dependent effects on apoptosis, differentiation, and cellular proliferation, mature BDNF primarily activates TrkB-associated survival pathways. ProBDNF/p75NTR-associated signaling has been linked to increased apoptosis and differentiation, as well as reduced cellular growth and migration, in human glioma specimens and experimental C6 glioma cells, demonstrating that the effects of neurotrophin signaling cannot be predicted solely by total BDNF expression [100]. Furthermore, TrkB appears in several isoforms, including full-length kinase-active TrkB and truncated variations like TrkB.T1, which lack the entire intracellular tyrosine kinase domain and may perform different signaling tasks. When assessing BDNF-associated responses, it is necessary to evaluate the relative expression and functional contributions of these isoforms in distinct glioma populations. As a result, enhanced BDNF expression should not always be considered therapeutically advantageous. Experiments assessing both neuronal and oncological outcomes in the same tumor-bearing paradigm, along with characterization of receptor expression and pertinent BDNF isoforms, are necessary to substantiate claims that BDNF augmentation protects neurons without concurrently promoting tumor growth. Overall, these findings suggest that generalized BDNF augmentation should not be seen as an intrinsically helpful therapeutic strategy in GBM. Instead, the major goal should be to maintain neuroprotective signaling while inhibiting malignant cell survival and development. Although BDNF-associated antioxidant interventions remain neuroprotective, their practical implementation in GBM requires a thorough assessment of tumor-promoting hazards, cell-specific responses, and combinations with established anticancer therapies.

## 5. Antioxidants as Modulators of Brain-Derived Neurotrophic Factor and Glioma Biology

Natural antioxidants, particularly plant-derived polyphenols, have attracted considerable interest because of their redox-modulating properties and potential antiglioma activities. Preclinical investigations have demonstrated that selected antioxidant compounds can influence glioma cell survival and treatment sensitivity through mechanisms involving oxidative stress and intracellular signaling pathway [101,102,103,104]. Separately, certain polyphenols have been associated with changes in BDNF expression and neurotrophic signaling in non-neoplastic experimental models [26]. However, these independent observations do not establish that BDNF/TrkB signaling directly mediates the antiglioma effects of antioxidant compounds. Therefore, the proposed interactions between antioxidant exposure, BDNF/TrkB signaling, and glioblastoma progression should be considered potential mechanistic relationships requiring direct functional validation.

Because this is a narrative review, the evidence described here comes from research conducted in various biological and clinical contexts and should be interpreted based on the source and directness of the available literature. To ensure clarity, the review papers were categorized into five evidence groups: (i) human/glioma/GBM research, including examination of BDNF/TrkB expression in patient tumor specimens and limited clinical or pharmacokinetic investigations of antioxidant agents [87,105]; (ii) preclinical glioma/GBM research conducted on tumor cell lines, glioma stem-like cells, or animal models [101,102,106,107]; (iii) non-neoplastic neurological studies exploring antioxidant mediated BDNF or neuroprotective signaling [108,109,110,111]; (iv) mechanistic research on other cancer types that gives insight on the carcinogenic role of redox-related pathways or BDNF/TrkB [112,113]; (v) Translational ideas that emerged from combining these several sources of research. Furthermore, findings based on non-GBM neurological models and other tumors are presented to offer biological plausibility and mechanistic background, but they are not regarded as concrete proof of therapeutic BDNF modulation in GBM.

### 5.1. Natural Antioxidants in Glioma

Several natural antioxidants have demonstrated promising effects in GBM models:

For the interpretation of compound-specific findings, three complementary evidence categories were considered. First, direct GBM-BDNF evidence refers to research that examine BDNF/TrkB signaling and glioblastoma-related biological outcomes in the same experimental or clinical setting. Second, GBM anticancer data without direct BDNF validation includes studies that show antioxidant-mediated effects on glioblastoma cell proliferation, apoptosis, migration, invasion, or treatment sensitivity but do not confirm the involvement of BDNF signaling. Examples include studies on curcumin-mediated temozolomide sensitization, EGCG-mediated inhibition of glioma stem-like cells, and quercetin-mediated suppression of IL-6/STAT3 signaling in glioblastoma cells [101,102,103,104]. Third, indirect mechanistic evidence refers to data from non-GBM neurological, oncological, or epidemiological models that have biological plausibility but do not show BDNF-mediated therapeutic action in glioblastoma. For instance, in experimental models of stress-induced depression, hydroxytyrosol-mediated activation of BDNF/TrkB/CREB signaling has been shown, and in experimental models of autoimmune encephalomyelitis and post-traumatic stress disorder, respectively, vitamin C- and vitamin E-associated neuroprotective effects have been studied [109,110,111]. These data suggest possible connections between neurotrophic signaling and antioxidant activity, however it is unclear how these findings relate to glioblastoma. Similarly, quercetin has shown anticancer efficacy in glioblastoma cell cultures, but direct evidence for BDNF-dependent pathways underlying these effects is limited [104]. As a result, findings from non-GBM models should be considered hypotheses rather than direct evidence for BDNF-targeted therapeutic success in glioblastoma. It is important to distinguish between direct evidence that BDNF/TrkB signaling promotes anticancer action in glioblastoma and changes in BDNF expression linked to antioxidants. Observing that a drug reduces glioma growth in one experimental type while modifying BDNF expression in another does not show a causal link between these findings. Mechanistic validation necessitates functional manipulation of BDNF or TrkB within the same experimental system, such as BDNF knockdown or overexpression, NTRK2/TrkB gene silencing, selective receptor inhibition, or rescue experiments that show that restoring BDNF/TrkB activity alters the observed therapeutic response. Experimental studies have shown that BDNF suppression inhibits glioma cell proliferation, migration, and invasion, whereas BDNF restoration reverses the effects of miR-489-3p in glioblastoma cell models [90]. Furthermore, genetic and pharmacological manipulation of TrkB has revealed its role in malignant neuron-glioma connections and tumor progression in experimental glioma models [25]. However, these findings do not establish that the antitumor effects of individual antioxidant compounds are mediated through BDNF/TrkB signaling. Accordingly, the proposed involvement of BDNF in antioxidant-mediated glioblastoma suppression should remain a mechanistic hypothesis unless supported by direct functional evidence.

#### 5.1.1. Curcumin (From Turmeric)

In experimental glioblastoma (GBM) models, curcumin, a naturally occurring polyphenol produced from Curcuma longa, has shown anticancer, anti-inflammatory, and antioxidant properties. Curcumin has been shown in preclinical studies to reduce glioma cell proliferation, induce apoptosis, and affect molecular pathways related with tumor survival and therapy resistance. Curcumin was found to improve temozolomide (TMZ)-induced cytotoxicity in U87MG glioblastoma cells and xenograft mice models. The combination treatment increased reactive oxygen species (ROS) formation, induced apoptosis, and inhibited AKT/mTOR signaling more efficiently than either alone [103]. These results indicate that curcumin may act as a chemo sensitizing agent under certain experimental conditions. However, the reported effects are still preclinical and cannot be readily construed as evidence of improved treatment results in individuals with GBM.

The clinical evidence for curcumin in glioblastoma is limited. A small preliminary clinical trial was undertaken with 13 glioblastoma patients, 10 of whom completed the study. Participants were given 70 mg of micellar curcuminoids orally three times per day for four days before the planned tumor excision. The formulation included curcumin, demethoxycurcumin, and bisdemethoxycurcumin. The researchers found a mean intratumoral curcumin content of 56 pg/mg tissue (9–151 pg/mg) and a mean serum concentration of 253 ng/mL (129–364 ng/mL). A statistically significant increase in intratumoral inorganic phosphate was found (*p* = 0.034), indicating potential changes in tumor energy metabolism. The study’s limitations were a small sample size, a short treatment duration, and an exploratory approach. Importantly, it did not show tumor regression, improved progression-free survival, overall survival, or increased chemoradiotherapy efficacy. As a result, these data focus on observable intratumoral exposure following oral micellar curcuminoid delivery, rather than clinical anticancer effectiveness [105].

More recently, Holdhoff et al. (2026) [114] reported preliminary findings from a Phase I/II clinical trial (NCT05768919) investigating intravenous liposomal curcumin combination with radiation and TMZ in patients with newly diagnosed high-grade gliomas. The data, presented at the 2026 American Society of Clinical Oncology Annual Meeting, covered 25 treated patients, all of whom had glioblastoma or molecular glioblastoma, IDH-wildtype, WHO grade 4. The study identified 350 mg/m^2^ as the recommended Phase II dose. Four patients (16%) reported treatment-related grade 3 or higher toxicities, which included neurological, haematological, and thromboembolic adverse reactions. Although preliminary findings revealed potential disease control, the study focused mostly on dose selection, safety, and treatment practicality. Its nonrandomized methodology and short follow-up preclude conclusions about increases in overall survival, progression-free survival, or superiority over standard treatment [114]. Furthermore, these findings were given in a conference abstract and must be confirmed by a full peer-reviewed publication and well-controlled clinical trials. Overall, existing evidence suggests that curcumin-based formulations can obtain detectable intratumoral exposure and may alter molecular mechanisms important for glioblastoma treatment. Nevertheless, the existing clinical evidence is preliminary, and curcumin’s therapeutic efficacy in GBM has not been confirmed. Further randomized controlled trials are needed to evaluate the best formulations, optimum doses, long-term safety, potential interactions with TMZ and radiation, and clinically significant therapeutic effects. As a result, curcumin should be considered an exploratory candidate rather than a proven supplementary treatment for glioblastoma.

#### 5.1.2. Resveratrol

Resveratrol exhibits antioxidant, antiproliferative, and proapoptotic effects in glioma cells. Its antiproliferative qualities have been investigated in various cancer types since Jang et al. first reported its antitumor potential in 1997 [115]. Research on nervous system cancers has focused primarily on gliomas, with findings suggesting potential therapeutic applications for improving the poor prognosis associated with these tumors. Zhang et al. report that resveratrol may induce cell cycle arrest in rat C6 glioma cells, accompanied by increased cell death and S-phase accumulation, with caspase-3 serving as a mediator. The precise mechanisms underlying this antitumor activity remain unclear, although several potential mechanisms have been investigated in in vitro experiments. Wang et al. report that resveratrol reduces the expression of oncogenic microRNAs (miRs) that are overexpressed in glioma cell lines and promote glioma development. After resveratrol treatment, the expression of miR-21, miR-19, and miR-30a-5p decreases in glioma cells, accompanied by enhanced expression of the tumor-suppressor genes PTEN and P53. Resveratrol may reduce EGFR overexpression in gliomas by inhibiting miR-21, implicated in antiapoptotic signaling and carcinogenesis [106]. Resveratrol may also modulate BDNF-related neurotrophic signaling, potentially accounting for its effects on oxidative stress, neural responses, and cell-survival pathways in the tumor microenvironment [116]. Mechanistic studies show decreased STAT3 expression and nuclear translocation, reduced Cyclin D1, increased LC3 and Beclin1, and extensive apoptosis, particularly in perivascular regions, indicating concurrent activation of apoptosis and autophagy with STAT3 pathway disruption. In an orthotopic rat glioblastoma model, resveratrol administered through lumbar puncture achieved increased intracranial drug availability by delivering the compound into the cerebrospinal fluid, thereby bypassing the conventional blood–brain barrier entry route. This administration approach was associated with reduced tumor growth and prolonged survival in treated animals. The observed antitumor effects were accompanied by suppression of STAT3 signaling, increased apoptosis, and enhanced autophagy [107]. However, these findings do not demonstrate improved blood–brain barrier penetration following systemic administration or establish BDNF/TrkB-dependent antitumor activity.

#### 5.1.3. Epigallocatechin-3-Gallate, the Main Catechin in Green Tea

Inhibits tumor growth and enhances the efficacy of temozolomide. Epigallocatechin-3-gallate (EGCG) reduces glioma stem-like cell properties by decreasing cell viability, neurosphere formation, migration, and survival signaling. It induces apoptosis by downregulating p-Akt and Bcl-2 and increasing PARP cleavage. EGCG inhibits P-glycoprotein (ABCB1) expression, thereby overcoming drug resistance and increasing the susceptibility of glioma stem-like cells to temozolomide [101]. EGCG enhances the anticancer efficacy of temozolomide by targeting the ER stress response. EGCG inhibits GRP78/BiP, a pro-survival ER chaperone associated with chemoresistance in GBM, thereby increasing tumor susceptibility to TMZ. In preclinical glioma models, EGCG combined with TMZ significantly prolonged survival compared to TMZ alone and reduced TMZ-induced GRP78 expression. Mechanistic studies report that GRP78 inhibition increases TMZ sensitivity in GBM cells, identifying the GRP78/CHOP-mediated ER stress pathway as a key target of EGCG-mediated chemosensitization [102]. Furthermore, EGCG is linked to modulation of BDNF/TrkB signaling in neurological models, suggesting that its antioxidant and neuroprotective effects may interact with BDNF-mediated pathways involved in cellular survival and stress responses [108].

#### 5.1.4. Hydroxytyrosol and Other Polyphenols Such as Quercetin

Hydroxytyrosol (HT), a key phenolic compound present in olives and olive oil, alongside other polyphenols, such as quercetin, has attracted attention for its potential anti-glioblastoma effects. These compounds can reduce oxidative stress and inflammation while suppressing tumor cell proliferation and promoting apoptosis. Their ability to modulate pathways, such as Nrf2, NF-κB, PI3K/Akt, and STAT3, suggests potential relevance to glioblastoma and neurodegenerative disorders [117]. HT demonstrates a favorable safety profile in animal studies, with minimal adverse effects. In rats, 13 weeks of daily oral HT administration show no toxicologically relevant effects, and a no-observed-adverse-effect level (NOAEL) of 500 mg/kg/day is proposed [118]. HT has demonstrated neuroprotective effects in a chronic unpredictable mild stress mouse model, where its administration was associated with reduced oxidative stress and neuroinflammation, together with increased hippocampal BDNF expression and phosphorylation of TrkB and CREB [109]. These observations support the involvement of BDNF-associated neurotrophic responses in a non-neoplastic neurological setting. However, they do not provide direct evidence that HT-mediated regulation of BDNF/TrkB signaling influences glioblastoma proliferation, progression, or therapeutic sensitivity. Therefore, the potential involvement of BDNF/TrkB signaling in the antiglioma effects of HT remains a hypothesis requiring direct mechanistic validation in GBM-specific experimental models.

#### 5.1.5. Quercetin

Quercetin is a naturally occurring flavonoid abundant in fruits, vegetables, and other plant-based foods, exhibiting high antioxidant, anti-inflammatory, and anticancer properties. Quercetin has attracted considerable interest as a potential therapeutic agent in cancer. Its multifaceted mechanisms of action have been investigated across various brain tumors, such as glioblastoma, glioma, neuroblastoma, astrocytoma, and medulloblastoma [119]. In glioblastoma models, quercetin inhibits cell viability, migration, and invasion and exerts anticancer effects by modulating signaling pathways involved in proliferation, apoptosis, autophagy, and cellular invasion, including the GSK3β/β-catenin/ZEB1 axis [119]. Quercetin has demonstrated inhibitory effects on glioblastoma cell growth and migration through mechanisms involving inflammatory and survival-associated signaling pathways, including IL-6/STAT3 signaling. Quercetin also inhibits GBM cell growth and migration by suppressing the IL-6-mediated JAK1/STAT3 signaling pathways [104]. However, these experimental observations do not establish a direct mechanistic relationship between quercetin exposure and BDNF/TrkB-dependent regulations of glioblastoma progression. Although interaction quercetin-associated signaling responses and neurotrophic pathway may be biologically plausible, the involvement of BDNF/TrkB signaling in quercetin-mediated antiglioma activity remains insufficiently investigated and requires further functional validation.

Vitamins C and E function as conventional ROS scavengers; however, their effects on brain tumors are generally less pronounced and more supportive than those of polyphenols [120]. Overall, 15 studies involving 3608 patients with glioma and 771,930 participants were included in the final analysis. The pooled analysis revealed that the highest vitamin C intake was associated with a significantly lower risk of glioma than the lowest intake (RR = 0.78; 95% CI: 0.63–0.96; *p* = 0.022). No significant associations were observed between vitamin A or vitamin E intake and glioma incidence (*p* > 0.05). Subgroup analyses of population-based case–control studies revealed an inverse correlation between vitamin C intake and glioma risk (RR = 0.82; 95% CI: 0.68–1.00, *p* = 0.049) with study quality (RR = 0.52, 95% CI: 0.29–0.92, *p* = 0.025) [121]. Beyond its antioxidant properties, vitamin C may also contribute to neuroprotective pathways involving BDNF signaling. In a multiple sclerosis model, combined vitamin A and C treatment reduces oxidative stress, inflammation, glial activation, and apoptosis while increasing BDNF and NeuN expression, suggesting that antioxidant-mediated BDNF signaling may contribute to neuronal survival and tissue repair. However, the direct anticancer effects of BDNF-related alterations on GBM remains uncertain [110]. A study shows that vitamin E prevents the decline in BDNF levels observed after PTSD-like stress, suggesting that its antioxidant activity may help preserve BDNF-mediated neuroprotective signaling and thereby reduce stress-induced memory impairment. These findings suggest a link between oxidative stress, epigenetic regulation, and BDNF signaling, and indicate that vitamin E may protect against PTSD-associated cognitive deficits by reducing oxidative damage while preserving BDNF expression and histone acetylation [111]. Overall, these findings suggest that vitamins C and E may modulate BDNF-associated neuroprotective pathways through antioxidant effects, although further research is needed to determine the direct therapeutic implications of this interaction in glioma and GBM. These compounds are particularly appealing because of their low toxicity and potential for long-term use as adjuncts to drug development.

The evidence concerning antioxidant compounds should be interpreted according to the clinical or experimental question addressed. Observational studies investigating the relationship between habitual dietary antioxidant consumption and eventual disease incidence focus on disease-risk relationships rather than the therapeutic efficacy of antioxidant therapies [122]. In contrast, studies examining glioblastoma cell proliferation, tumor development, apoptosis, and temozolomide sensitivity focus on experimental anticancer activity [102,103,123]. Early clinical trials involving glioblastoma patients also investigated tumor exposure to orally administered curcuminoids and the possibility of pharmacological ascorbate combined with normal chemoradiotherapy, but these investigations could not show conclusive therapeutic efficacy [105,124]. Studies on neuroprotection or cognitive function in non-GBM populations give indirect evidence for prospective supportive-care uses, but should not be taken as proving tumor control or improved treatment results in glioblastoma. Accordingly, prevention-related observations, experimental tumor-treatment findings, and supportive-care hypotheses should be distinguished according to their respective clinical relevance, study design, and level of evidence.

It is important to note that the BDNF/TrkB-related results and antiglioma activity summarized in Table 1 are not necessarily causally connected pathways. BDNF-associated benefits for various antioxidant compounds were observed predominantly in non-GBM neurological models, while antiproliferative or treatment-sensitizing properties were demonstrated separately in experimental glioma models. As a result, the combination of these data should not be construed as proof that BDNF/TrkB signaling is directly involved in antioxidant-induced tumor suppression. Direct mechanistic validation through BDNF/TrkB genetic manipulation, receptor inhibition, or rescue experiments remains necessary to establish such relationships.

### 5.2. Mechanisms of Action

Antioxidants regulate glioma biology and BDNF signaling through multiple interconnected mechanisms.

#### 5.2.1. Direct Reactive Oxygen Species Scavenging

Polyphenols directly neutralize ROS, thereby reducing oxidative stress and protecting cellular components [129]. This contributes to redox homeostasis and may inhibit ROS-induced proliferation, invasion, and angiogenesis in GBM cells while protecting normal neurons from oxidative stress. Direct radical-scavenging activity is particularly relevant to the brain, which is highly susceptible to oxidative damage owing to its high metabolic activity and relatively limited antioxidant defenses [130]. However, the biological effects of polyphenols should not be interpreted as uniformly antioxidant or beneficial in glioblastoma. Depending on the compound, concentration, and cellular context, polyphenols may influence oxidative stress through antioxidant or pro-oxidant mechanisms. These effects may involve direct radical scavenging, modulation of endogenous antioxidant systems, mitochondrial responses, and redox-sensitive signaling pathways [26]. Nevertheless, whether these mechanisms contribute to glioblastoma suppression or tumor-cell adaptation must be determined using compound-specific experimental evidence.

#### 5.2.2. Potential Regulation of BDNF-Associated Signaling Through CREB and Nrf2 Pathways

**Figure 3:**
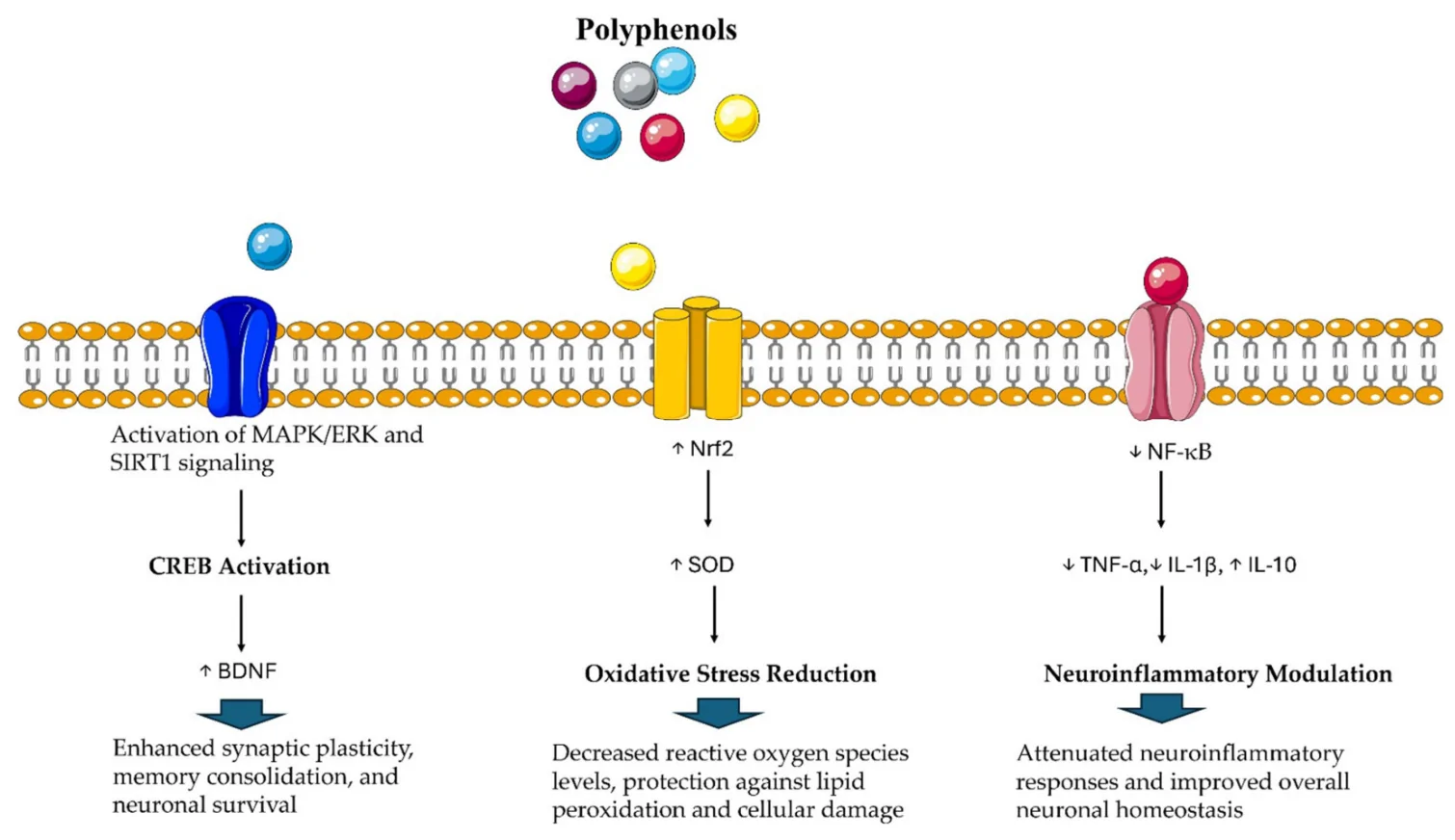
Polyphenols contribute to neuroprotection, antioxidant defense, and regulation of neuroinflammatory processes through multiple biomolecular pathways. The pathways shown represent general antioxidant, anti-inflammatory, and neuroprotective mechanisms associated with polyphenols. Their depiction does not establish that these effects occur simultaneously in glioblastoma or that BDNF/TrkB signaling mediates the antitumor effects of polyphenols in GBM [131]. Servier Medical Art is licensed under the Creative Commons Attribution 3.0 Unported License (https://creativecommons.org/licenses/by/3.0/; retrieved 8 September 2026).

Selected polyphenols have been reported to influence CREB-associated transcriptional responses and BDNF expression in non-neoplastic neurological models. Separately, polyphenols have been investigated for their effects on Nrf2-dependent antioxidant defenses, inflammatory responses, and redox-sensitive signaling pathways [26,131]. Although these observations suggest potential biological interactions among antioxidant exposure, CREB activation, BDNF/TrkB signaling, and Nrf2-mediated antioxidant regulation, direct evidence demonstrating that these pathways act together to mediate the antiglioma effects of polyphenols remains insufficient. Moreover, Nrf2 activation may have different biological consequences in nonmalignant neural cells and glioblastoma cells, where enhanced antioxidant defenses may support tumor survival and therapeutic resistance. Consequently, the proposed CREB-BDNF/TrkB-Nrf2 relationship should be interpreted as a potential mechanistic interaction rather than an experimentally validated pathway responsible for antioxidant-mediated glioblastoma suppression [131]. Polyphenols have been investigated for their anti-inflammatory, antioxidant, and neuroprotective activities in multiple experimental contexts (Figure 3). They modulate key signaling pathways involved in oxidative stress, neuronal survival, and mitochondrial function. For example, polyphenols can enhance the expression of antioxidant enzymes and protect neurons from oxidative damage by modulating the Nrf2 pathway [131].

#### 5.2.3. Epigenetic Modifications

Polyphenols modulate epigenetic processes by inhibiting DNA methyltransferases and histone deacetylases. This reactivates tumor suppressor genes and downregulates oncogenes [132]. In gliomas, these alterations can reduce stemness and increase tumor cell susceptibility to therapy. These epigenetic modifications may also affect BDNF expression and genes involved in neuroplasticity [91]. However, therapeutic strategies targeting neurogliomal circuits may provide a promising approach for selectively targeting brain tumors while preserving normal brain tissue. Potential targets include AMPA/NMDA receptor antagonists, chloride transporter inhibitors, synaptogenic protein blockers, and agents that modulate tumor microtubule dynamics. Validation, accurate patient classification, and integration into multidisciplinary neuro-oncology procedures remain necessary for the clinical application of these approaches. Recognizing brain cancer as a disease of pathological neuroplasticity rather than solely as a proliferative malignant neoplasm creates new research opportunities for developing therapies that disrupt electrical and synaptic communication between glioma cells and neurons, potentially improving treatment outcomes for these tumors [133].

#### 5.2.4. Exercise-Associated Neuroplasticity and Potential Combined Effects with Polyphenols

Physical exercise and polyphenol exposure have independently been associated with changes in neurotrophic, inflammatory, and redox-sensitive pathways. Physical exercise has been shown to regulate hippocampal BDNF expression through the PGC-1α/FNDC5 pathway and through exercise-associated metabolic and epigenetic mechanisms involving β-hydroxybutyrate and histone deacetylase regulation [134,135]. Although these findings confirm the neuroplasticity-related effects of exercise, they were not achieved in glioblastoma-bearing experimental models and do not demonstrate anticancer action. Furthermore, the observation that exercise and polyphenols separately alter BDNF signaling or oxidative stress in GBM does not support a synergistic therapeutic relationship. In order to establish synergy, untreated controls, exercise alone, polyphenol therapy alone, and their combination must be experimentally compared. Additionally, the interaction between interventions must be appropriately statistically assessed [136]. In addition, in experimental glioma models, BDNF/TrkB signaling may also encourage malignant synaptic plasticity and glioma growth. Therefore, before claiming synergistic antitumor or neuroprotective benefits, future research should assess both tumor progression and cognitive outcomes within the same molecularly characterized glioblastoma-bearing models. Exercise-polyphenol combinations should therefore be regarded as exploratory.

**Figure 4:**
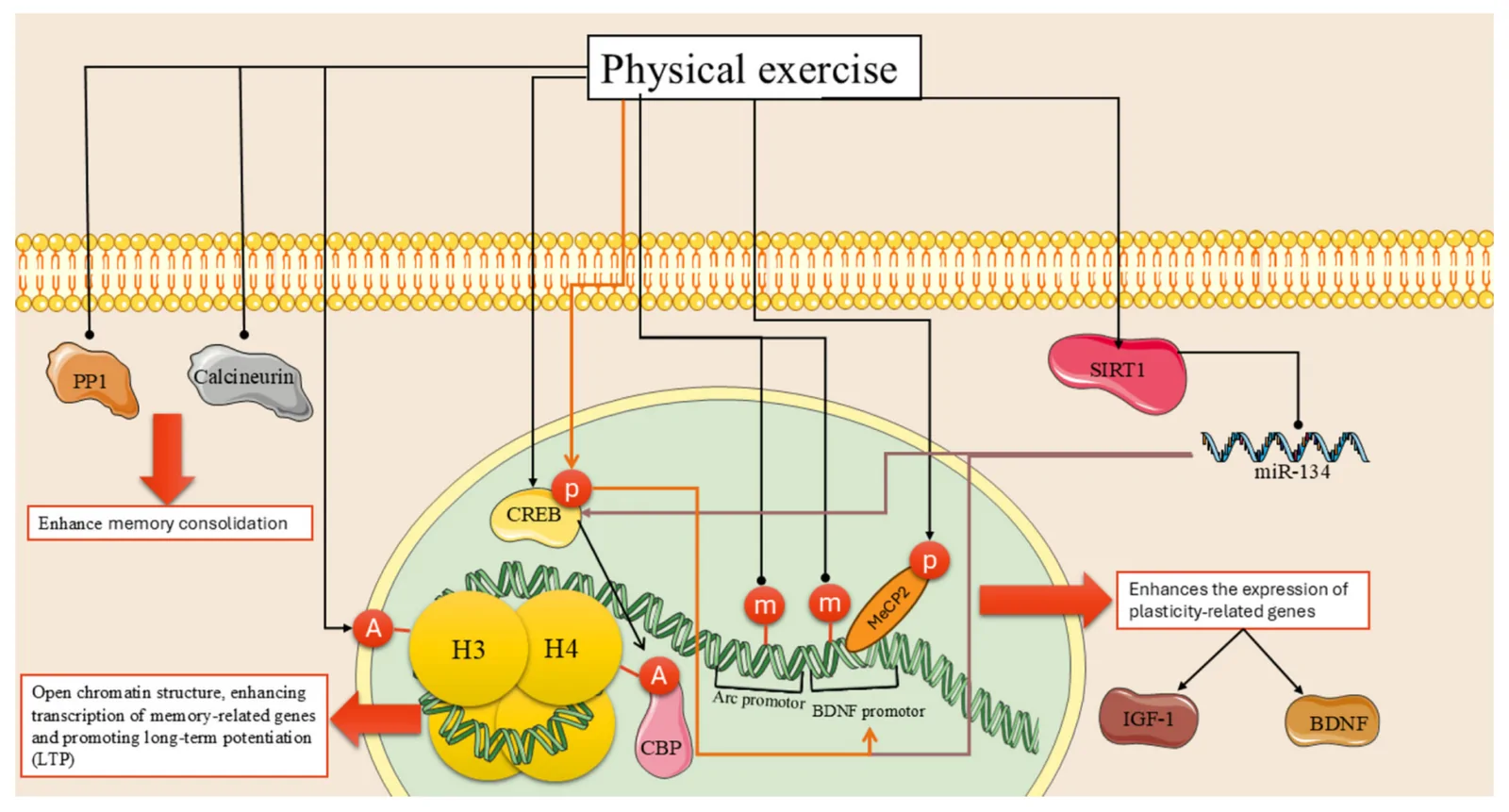
Proposed epigenetic pathways underlying exercise-induced neuroplasticity. Physical activity modulates CREB, SIRT1, DNA methylation, histone acetylation, MeCP2, and miR-134 signaling, potentially enhancing BDNF and IGF-1 levels and improving synaptic plasticity and memory consolidation. The illustrated epigenetic and neurotrophic pathways are derived primarily from non-neoplastic experimental contexts and provide a conceptual framework for understanding exercise-associated neuroplasticity. They should not be interpreted as demonstrating enhanced tumor control or BDNF-mediated therapeutic efficacy in glioblastoma. Adapted from [138] under CC BY 4.0 license (https://creativecommons.org/licenses/by/4.0/). Abbreviations: BDNF—brain-derived neurotrophic factor; CREB—cAMP response element-binding protein; IGF-1—insulin-like growth factor 1; LTP—long-term potentiation; MeCP2—methyl-CpG-binding protein 2; miR-134—microRNA-134; PP1—protein phosphatase 1; SIRT1—sirtuin 1.

Physical activity is one of the most potent physiological inducers of BDNF expression, and polyphenol supplementation may further enhance this effect [137]. Exercise increases BDNF expression via PGC-1α and FNDC5, while polyphenols enhance BDNF signaling, support mitochondrial function, and exert anti-glioma effects. Exercise also induces FNDC5 expression in the hippocampus at levels quantitatively comparable to those reported in skeletal muscle. Its induction is quantitatively comparable to that of BDNF, a neurotrophic mediator of exercise in the brain, and to that of cFOS, ARC, and ZIF268, key markers of neuronal activity [134]. Although exercise and selected polyphenols have independently demonstrated effects on neurotrophic signaling and cellular stress responses, the cited study do not establish that their combination reduces glioblastoma tumor growth and cognitive impairment in the same tumor bearing experimental models. Accordingly, potential combined benefits require further investigation using appropriately controlled experimental designs [103,134,138]. Physical exercise may complement therapeutic interventions by enhancing epigenetic and transcriptional processes supporting brain development and cognitive function. Exercise increases CREB activation, histone acetylation, and BDNF transcription while modulating DNA methylation, MeCP2, SIRT1, and miR-134 signaling. These changes may increase the expression of neuroplasticity-related proteins, such as BDNF and IGF-1, while inhibiting memory-repressive pathways involving PP1 and calcineurin. Collectively, these pathways may improve synaptic plasticity, memory consolidation, and cognitive resilience, supporting exercise as a potential adjunctive therapy for brain tumors [138]. Exercise represents a potential supportive-care intervention whose safety and benefits in glioblastoma require disease-specific evaluation. Figure 4 illustrates the proposed epigenetic pathways underlying the beneficial effects of physical exercise on neural plasticity and cognitive performance.

### 5.3. Evidence from Preclinical Models and Human Studies

#### 5.3.1. Preclinical Evidence

The efficacy of these antioxidants is supported by several in vitro and in vivo studies. In GBM cell lines (U87 and U251), curcumin and resveratrol induce cell-cycle arrest and apoptosis and reduce invasion [139]. EGCG increases temozolomide sensitivity in mouse models. Separate experimental studies have investigated the antiglioma effects of antioxidant compounds and the neuroplasticity-related effects of exercise. However, the cited evidence does not establish that combined treatment improves both tumor control and cognitive function in the same glioblastoma model [44,102,138]. Nanodelivery techniques, such as nanocurcumin, increase bioavailability and brain penetration. Combination therapy with curcumin-loaded NPs may reduce the required dose of the primary therapeutic agent, potentially improving therapeutic efficacy while reducing systemic toxicity. Furthermore, while functionalized nanoparticles can enhance targeted drug delivery, their nanoscale size and high surface area may promote particle aggregation and limit drug loading capacity [140].

#### 5.3.2. Human Evidence from Non-GBM Populations and Limited Glioma Studies

Although natural antioxidants and polyphenols have shown potential benefits in experimental glioblastoma models, there is inadequate clinical evidence to suggest their therapeutic efficacy in GBM patients. Bondonno et al. studied 55,985 adults from the Danish Diet, Cancer, and Health cohort to determine the relationship between habitual dietary flavonoid intake and incident dementia. However, this was an observational study looking into dementia risk rather than a clinical trial evaluating antioxidant supplementation or cancer treatment. Moreover, the study did not evaluate glioma development, treatment response, or cancer-related cognitive impairment. As a result, its findings give epidemiological background for dietary flavonoids and neurological health but do not show the therapeutic efficacy of antioxidant therapies in GBM [122]. Similarly, Huang et al. conducted a 12-week, randomized controlled experiment to assess an antioxidant-focused dietary intervention in 100 lung cancer patients suffering from cancer-related cognitive impairment. The intervention was associated with improvements in cognitive performance, quality-of-life measures, and selected oxidative stress biomarkers compared with standard dietary counseling. The intervention improved Montreal Cognitive Assessment scores and glutathione and superoxide dismutase levels while reducing malondialdehyde levels, with a significant effect on cognitive function (Cohen’s d = 0.84). Although these findings indicate that antioxidant-focused dietary approaches may influence cognitive and oxidative stress outcomes in lung cancer patients, the trial excluded patients with glioma or GBM and did not look into tumor progression, survival, temozolomide sensitivity, or radiotherapy response in brain tumors. As a result, these findings cannot be regarded direct clinical evidence for antioxidant treatment in GBM [141]. The distinction between these populations is especially essential since the biological effects of antioxidant supplementation can vary significantly between normal brain tissue and malignant glioma cells. While reducing oxidative damage may have neuroprotective effects, antioxidant-mediated strengthening of cellular defense mechanisms could theoretically shield tumor cells from oxidative stress caused by conventional anticancer treatments.

Additionally, BDNF/TrkB signaling may help neurons survive while also increasing glioma cell proliferation and malignant synaptic plasticity. Overall, the present evidence promotes additional research into antioxidant-mediated redox regulation and BDNF-associated neuroprotection, but it does not demonstrate therapeutic benefit in GBM. Future randomized controlled trials involving glioblastoma patients are needed to determine optimum dosages, treatment timing, bioavailability, interactions with temozolomide and radiotherapy, and potential effects on tumor progression.

To provide a systematic comparison of available experimental and clinical evidence, the therapeutic relevance of selected antioxidants was assessed based on study design and level of evidence, which included in vitro investigations, animal studies, observational human research, early clinical investigations, and GBM-specific clinical studies (Table 2). The classification differentiates between experimentally established anticancer activity and clinically validated treatment effects.

As demonstrated in Table 2, clinical research on antioxidant-based therapies in glioblastoma is limited and varied. A presurgical study using micellar curcuminoids found substantial intratumoral exposure but no improvement in treatment results. More recently, a randomized Phase II trial of a seven-component nutraceutical product containing curcumin among 53 glioblastoma patients found no statistically significant improvement in overall survival over placebo (14 versus 13 months; *p* = 0.752). Similarly, an exploratory presurgical clinical study combining resveratrol and copper found alterations in tumor-associated molecular and immunological indicators but did not show clinical survival advantages. Pharmacological ascorbate has advanced to Phase I clinical trials in combination with radiotherapy and temozolomide, demonstrating preliminary feasibility and tolerability; however, the uncontrolled study design did not allow for definitive conclusions about therapeutic superiority or survival improvement. Overall, these findings suggest that the therapeutic efficacy of antioxidant-based therapies is not well established, and that good results from experimental models should not be easily transferred to patients with GBM.

Importantly, the evidence summarized in Table 1 and Table 2 addresses distinct objectives, including experimental glioblastoma treatment, observational associations with disease risk or neurological outcomes, and potential supportive-care applications. These conditions should not be considered equivalent proof of therapeutic efficacy. Furthermore, differences in animal models, compound concentrations, administration routes, treatment durations, and assessed results prevent direct comparisons between research. Therefore, findings should be interpreted according to their respective experimental contexts, and clinical recommendations should not be inferred from preclinical or non-GBM evidence alone.

Natural antioxidants, particularly polyphenols, such as curcumin, and EGCG, have demonstrated effects on oxidative stress, redox-sensitive signaling, and glioma-associated biological responses in different preclinical models [101,102,103]. Separately, selected polyphenols have been associated with BDNF-related neurotrophic responses in non-neoplastic neurological systems [26,131]. However, the available evidence does not establish that antioxidant-associated modulation of BDNF/TrkB signaling directly mediates glioblastoma suppression or treatment sensitization. Proposed interactions involving ROS regulation, CREB/Nrf2 signaling, epigenetic mechanisms, and neurotrophic pathways therefore require direct experimental validation within the same glioblastoma model. Further mechanistic and clinical investigations are necessary to determine whether these interactions can be therapeutically targeted without promoting tumor survival or compromising treatment efficacy.

## 6. Therapeutic Potential and Clinical Evidence

Natural antioxidants are being investigated as potential adjunctive approaches in glioblastoma because of their effects on oxidative stress and other cellular signaling pathways; however, BDNF/TrkB-dependent mediation of their therapeutic activity remains insufficiently established. While antioxidant compounds have been studied as possible radiosensitizers, chemosensitizers, and neuroprotective agents, the quality of the evidence for these uses varies significantly. Antioxidant-related neuroprotective effects seen in non-GBM neurological models cannot prove cognitive preservation in patients receiving GBM treatment, and experimental improvement of temozolomide-associated cytotoxicity or radiation responsiveness does not necessarily translate into improved clinical survival. Furthermore, there is yet limited evidence regarding the safety and therapeutic effectiveness of combining these substances with immunotherapy. Accordingly, these proposed applications should be regarded as investigational until supported by appropriately designed, controlled GBM-specific clinical trials with predefined oncological and neurocognitive endpoints [102,103,124,142].

### 6.1. Antioxidants as Adjunctive Therapies

Antioxidants may act as both radiosensitizers and chemosensitizers while providing neuroprotection [147]. Polyphenols, such as curcumin and EGCG, may increase ROS levels and disrupt survival pathways, including NF-κB and STAT3, potentially sensitizing GBM cells to temozolomide and radiation [125,148]. These compounds may also protect normal neurons and glial cells from treatment-induced oxidative damage, potentially reducing neuroinflammation and cognitive impairment [119]. This dual activity may enhance tumor-cell killing while protecting normal brain tissue, offering a potential benefit over conventional therapies.

### 6.2. Combination Therapies

Polyphenols and Exercise: Physical exercise and selected polyphenols have independently been investigated for their effects on neurotrophic signaling, oxidative stress, inflammatory responses, and cellular metabolism. Exercise-associated increases in BDNF expression and neuroplasticity have been reported predominantly in non-neoplastic experimental settings [134,135]. Whereas, curcumin, resveratrol, and other polyphenols’ antiglioma properties have typically been studied in different tumor models [102,103,107,123,145]. The cited evidence does not establish a formally demonstrated synergistic interaction between exercise and polyphenol supplementation for glioblastoma tumor control or cognitive preservation. Specifically, subsequent improvements in neurocognitive and oncological outcomes in the same molecularly characterized GBM-bearing mice have not been sufficiently verified. Demonstrating synergy would require controlled experimental comparisons of exercise alone, polyphenol treatment alone, and their combination, together with appropriate assessment of treatment interactions [136]. Therefore, combined exercise-polyphenol interventions should be regarded as exploratory strategies requiring controlled factorial experiments, assessment of tumor progression and cognitive outcomes, and investigation of potential BDNF/TrkB-mediated tumor-promoting effects [25].

#### Nanodelivery Systems

Natural antioxidants have limited bioavailability and BBB penetration [149]. Advanced nanodelivery technologies, including liposomal curcumin, PLGA nanoparticles, and BBB-targeted nanoformulations, may enhance brain delivery [149,150]. These approaches may reduce toxicity by improving bioavailability, enabling controlled release, and reducing the effective dose. Nanocarrier-based formulations of curcumin have been investigated as potential strategies to improve its solubility, stability, bioavailability, and delivery to glioblastoma cells [148]. Preclinical investigations using curcumin- and temozolomide-loaded magnetic nanoparticles demonstrated enhanced drug accumulation and cytotoxic effects in glioblastoma spheroid models [151]. In experimental models, localized nanogel formulations containing curcumin and temozolomide were tested for long-term drug release and suppression of temozolomide-resistant glioblastoma [152]. However, these data do not support clinical treatment efficacy or show that the reported antiglioma effects are mediated by BDNF/TrkB signaling. Further studies are required to determine tumor-specific drug distribution, pharmacokinetics, treatment response, and BDNF-associated molecular mechanisms in well-characterized glioblastoma models.

The biological effects of natural antioxidants in glioblastoma are determined by compound concentration, exposure time, cellular environment, and combinations with conventional anticancer therapies. At specific concentrations, antioxidant compounds may lower intracellular ROS and protect nonmalignant neurons from oxidative damage. However, similar cytoprotective actions in glioblastoma cells may improve tumor survival and diminish sensitivity to radiotherapy or chemotherapy [7,153,154]. Conversely, some polyphenols may exert pro-oxidant effects under specific experimental conditions, increasing oxidative damage and promoting tumor-cell apoptosis. Curcumin has been shown in experiments to increase temozolomide-associated cytotoxicity by inhibiting survival-related signaling pathways and generating more ROS. As a result, a compound’s therapeutic efficacy cannot be independently predicted by its antioxidant or pro-oxidant classification [7,153]. An additional aspect is the timing of antioxidant delivery in relation to temozolomide treatment and ionizing radiation. Because various treatments may entail oxidative stress-related cytotoxic processes, antioxidant exposure before to, during, or following therapy may result in diverse biological effects. Nrf2 knockdown has been linked to increased vulnerability to radiation-induced oxidative stress in experimental glioblastoma neurosphere models, underscoring the significance of endogenous antioxidant defenses in treatment responses [11].

Tumor-specific molecular characteristics may further influence therapeutic responses. While Nrf2 signaling can support cytoprotection in nonmalignant brain tissues, increased Nrf2 activity could enhance antioxidant defenses in malignant cells and contribute to treatment resistance [7,11,154]. Similarly, BDNF/TrkB signaling supports synaptic plasticity and neuronal survival, it may potentially stimulate the formation of malignant gliomas via tumor-associated neurotrophic and synaptic signaling pathways [25]. Thus, within the same experimental framework, subsequent studies should assess concentration-dependent responses, tumor versus normal-cell selectivity, treatment timing, Nrf2 activation status, and BDNF/TrkB expression.

### 6.3. Challenges and Limitations

Despite encouraging outcomes, several challenges must be resolved before broad clinical adoption:

#### 6.3.1. Dose Dependence

Antioxidant effects are biphasic. High doses may exert pro-oxidant effects or inadvertently promote tumor cell survival, while low-to-moderate concentrations may offer therapeutic benefits [155].

#### 6.3.2. Bioavailability

Limited oral absorption and rapid metabolism restrict the therapeutic efficacy of many natural polyphenols without advanced formulations [156]. Blood–Brain Barrier: Drug delivery to tumor sites is limited in patients with GBM when the BBB remains intact or only partially disrupted [157].

#### 6.3.3. Potential Pro-Tumorigenic Effects

Certain antioxidants may activate protective pathways, such as Nrf2, in tumor cells at inappropriate concentrations or in specific GBM molecular subtypes, potentially promoting treatment resistance rather than decreasing it [153].

These challenges highlight the need for tailored strategies in molecularly defined IDH-wildtype glioblastoma, including assessment of Nrf2 activity and other relevant tumor-specific characteristics. In conclusion, antioxidants, particularly polyphenols, may serve as adjuncts to conventional GBM therapy due to their neuroprotective properties, potential to enhance chemoradiation sensitivity, and interaction with exercise. Advances in nanodelivery systems may further improve their therapeutic utility. However, dosing, bioavailability, and context-dependent effects remain key challenges. Well-designed randomized clinical trials are needed to evaluate these approaches and establish their integration into existing treatment protocols.

## 7. Future Directions and Research Gaps

Despite advances in understanding oxidative stress, Nrf2 signaling, and BDNF functions in GBM, key gaps still limit the clinical application of antioxidant-based treatments. Future studies should move beyond primarily preclinical evidence toward individualized, mechanism-driven, and biomarker-guided approaches accounting for long-term outcomes, drug interactions, and tumor heterogeneity.

### 7.1. Personalized Antioxidant Approaches

Future GBM-specific investigations should prioritize molecularly informed experimental strategies involving tumors classified as IDH-wildtype under WHO CNS5, with exploratory assessment of Nrf2 activity, BDNF/TrkB expression, and other relevant molecular characteristics. IDH-mutant astrocytomas should be analyzed as a separate diagnostic entity rather than considered a molecular subgroup of glioblastoma. It should be mentioned that IDH-mutant astrocytomas are not classed as glioblastomas by WHO CNS5. As a result, comparisons based on IDH mutation status are meant to illustrate biological distinctions between different glioma types rather than implying that IDH-mutant tumors are a molecular subtype of glioblastoma [2]. Baseline BDNF and TrkB expression and Nrf2 activity may be investigated as exploratory molecular biomarkers in glioblastoma; however, their clinical utility for predicting responses to antioxidant-based or BDNF-modulating treatment has not been established. Future studies should evaluate these markers prospectively alongside validated clinical endpoints and established molecular characteristics. Integrated transcriptomics, proteomic, and genomic approach may help identify candidate response-associated signatures, but their predictive performance and clinical usefulness require independent validation in molecularly characterized glioblastoma cohorts. Combining transcriptomics, redox proteomics, and genomics may improve therapy response selection and patient stratification while reducing adverse effects [158].

### 7.2. Literature and Mechanistic Gaps

Despite encouraging findings for curcumin, resveratrol, EGCG, and other polyphenols, most evidence remains derived from in vitro and animal models. The interactions among antioxidant signaling, BDNF/TrkB, and Nrf2 within the GBM microenvironment remain poorly defined. The effects of individual antioxidants on these pathways in tumor cells versus normal neurons also remain unclear. Additionally, antioxidants warrant greater attention for their biphasic or hormetic effects because excessive activation of cytoprotective pathways, such as Nrf2, may promote tumor cell survival. Therefore, thorough dose–response studies are needed to determine treatment windows that maximize anticancer efficacy while minimizing adverse effects.

Drug delivery and bioavailability remain significant challenges. Nanodelivery approaches may improve the solubility, stability, and brain penetration of poorly bioavailable drugs; however, systematic comparisons of nanoformulations in orthotopic GBM models and assessments of their long-term safety remain limited. Furthermore, heterogeneity of GBM, including classical, mesenchymal, and proneural features, may substantially affect responses to antioxidant therapy, particularly in tumors with varying reliance on Nrf2 signaling.

### 7.3. Need for Rigorous Clinical Trials

Limited high-quality clinical evidence remains a major barrier to the translation of antioxidant-based interventions into glioblastoma treatment. Well-designed randomized controlled trials are required to assess the safety and efficacy of these interventions as adjuncts to standard-of-care therapy. Patient eligibility should be established according to WHO CNS5 diagnostic criteria, with glioblastoma defined as an adult-type IDH-wildtype diffuse glioma, CNS WHO grade 4. IDH-mutant astrocytomas should be considered diagnostically distinct and evaluated separately rather than included as a molecular subgroup of glioblastoma [2]. In contrast, BDNF/TrkB expression and Nrf2 activity should be evaluated as exploratory molecular or pharmacodynamic biomarkers rather than established criteria for treatment stratification. Their potential predictive value requires prospective validation and demonstration of clinically meaningful associations with treatment responses. Future studies should assess overall survival, progression-free survival, treatment-related toxicity, neurocognitive outcomes, and quality of life, while accounting for antioxidant dose, formulation, blood–brain barrier delivery, and possible interactions with temozolomide and radiotherapy. Where biologically justified, exploratory BDNF/TrkB and Nrf2 measurements may be incorporated to investigate mechanisms of response or resistance, but should not be interpreted as validated surrogate markers of therapeutic efficacy.

### 7.4. Integration with Immunotherapy and Targeted Therapies

Combining antioxidant strategies with emerging therapies represents a potential research direction. Polyphenols and Nrf2-regulated compounds may modulate immune-cell function, oxidative signaling, and the tumor microenvironment. They may also interact with oncolytic viruses, CAR T-cell treatments, and immune checkpoint inhibitors. Similarly, overlapping redox, survival, and stress-response pathways may support rational combinations with molecularly targeted strategies, such as EGFR- or PARP-directed therapies and antiangiogenic agents. However, antioxidant-mediated ROS suppression may interfere with treatments relying on oxidative stress for anticancer efficacy; therefore, these combinations require rigorous preclinical validation.

### 7.5. Prevention of Cognitive Decline in Glioblastoma Survivors

As survival rates improve, maintaining cognitive function and quality of life becomes increasingly crucial. Treatment-induced cognitive impairment can affect memory, attention, executive function, and daily functioning. Antioxidants enhancing BDNF signaling and reducing oxidative stress and neuroinflammation may provide neuroprotective effects. Therefore, future long-term research should assess the potential of structured exercise combined with BDNF-enhancing antioxidant therapies to prevent or reduce cognitive impairment during and after GBM therapy. These investigations should differentiate potential effects on tumor survival from neuroprotective effects in normal brain tissue.

## 8. Conclusions

The intricate interplay of oxidative stress, antioxidant defense mechanisms, and brain-derived neurotrophic factor (BDNF)/TrkB signaling is a key topic of study in glioblastoma (GBM) biology. Preclinical evidence suggests that natural antioxidants, particularly polyphenols like curcumin, resveratrol, and epigallocatechin-3-gallate, can modulate redox-sensitive signaling pathways, influence glioma cell proliferation and survival, and alter molecular mechanisms involved in therapeutic resistance. However, these findings are based primarily on in vitro research and animal models, and their clinical relevance is unknown. Importantly, the therapeutic implications of antioxidant-mediated BDNF regulation must be carefully considered, as BDNF/TrkB signaling has opposite biological consequences in normal neuronal and malignant glioma cells. Although BDNF promotes neuronal survival, synaptic plasticity, and tolerance to oxidative stress, abnormal activation of the same signaling pathways may enhance glioma cell proliferation, invasion, and treatment resistance. Furthermore, antioxidant-mediated strengthening of cellular defense mechanisms may prevent malignant cells from oxidative damage caused by conventional anticancer treatments. When assessing antioxidant-based therapy methods, these context-dependent effects emphasize the significance of differentiating neuroprotective responses from tumor-promoting processes. There is presently not enough high-quality clinical evidence to suggest that natural antioxidants increase overall survival, progression-free survival, cognitive outcomes, treatment tolerance, or therapeutic efficacy in patients with GBM, despite promising preclinical results. Clinical advantages for glioblastoma should not be inferred directly from observational research and clinical investigations with non-GBM populations. Furthermore, several translational issues remain unresolved, including limited bioavailability, blood–brain barrier penetration, optimal dosing, pharmacokinetic variability, potential interactions with temozolomide and radiotherapy, and tumor-specific differences in redox and BDNF-related signaling.

Future research should focus on well-characterized patient-derived glioblastoma models, clinically relevant in vivo studies, and appropriately designed GBM-specific clinical trials. Identifying molecular biomarkers that predict therapy responses, assessing cell-selective modulation of BDNF/TrkB signaling, and understanding if neuronal protection can be achieved without increasing tumor survival should all be prioritized. Although BDNF/TrkB signaling has been functionally implicated in glioma biology, the available compound-specific evidence does not establish that BDNF mediates antioxidant-induced tumor suppression. Future investigations should incorporate BDNF/TrkB loss-of-function and rescue experiments in molecularly characterized glioblastoma models before attributing anticancer effects to this pathway. Clinical trials should include oncological objectives such as tumor progression and overall survival, as well as neurological outcomes such as cognitive function, treatment-related toxicity, and quality of life. Collectively, natural antioxidants and strategies targeting the antioxidant–BDNF–redox axis represent experimental candidates worthy of further mechanistic and translational investigation. Their potential incorporation into glioblastoma management remains contingent upon rigorous demonstration of safety, tumor-specific activity, and clinically meaningful benefits in appropriately controlled studies.

## Figures and Tables

**Table 1:** Natural antioxidants relevant to GBM and the level of evidence supporting their effects on redox and BDNF-related pathways.

Antioxidant/Compound and Sources	Effects on Oxidative Stress/Redox System	BDNF/TrkB-Associated Findings and Neuro Neuroprotective Observation	Evidence Context Model/Level of GBM Relevance	Anticancer/Antiglioma Effects	Therapeutic Evidence	References
Curcumin Turmeric (Curcuma longa)	Reduces oxidative stress, modulates redox-sensitive signaling, and may regulate antioxidant responses associated with Nrf2.	May modulate BDNF-related signaling and support neuroprotective outcomes.	Human GBM exposure study; Glioma/GBM preclinical studies; neurological model for BDNF/Mixed: direct GBM+indirect BDNF evidence	Inhibits glioma growth, induces G2/M cell-cycle arrest, apoptosis, and autophagy, and may enhance responses to TMZ and radiation.	Limited/early human data; preclinical evidence from cell and animal models	[105,125,126,127,128]
Resveratrol Berries, red wine, and grapes	Modulates cellular redox signaling and oxidative stress.	May modulate BDNF-related neurotrophic and survival signaling.	Glioma cell lines and orthotopic animal GBM models; Direct antiglioma evidence; indirect BDNF evidence	Decreases glioma formation, modulates oncogenic miRNAs, increases PTEN/P53, suppresses STAT3, promotes autophagy and apoptosis, and decreases tumor volume in orthotopic models	Predominantly preclinical evidence	[106,107,115,116]
EGCG Green tea (Camellia sinensis)	Modulates ER stress and oxidative responses	Potential neuroprotective effects associated with BDNF/TrkB signaling regulation in neurological models	Neurological BDNF models, orthotopic GBM models, and glioma stem-like cells/ Direct antiglioma evidence; indirect BDNF evidence	Inhibits p-Akt/Bcl-2 signaling, promotes PARP cleavage, inhibits P-glycoprotein, reduces glioma stem-like characteristics, migration, and survival, and increases TMZ sensitivity.	Strong in vitro and animal evidence	[101,102,108]
HT Olives and olive oil	Reduces neuroinflammation and oxidative stress and modulates Nrf2-related pathways	Stimulates BDNF/TrkB/CREB signaling and increases BDNF expression in neurological models	limited research on brain tumours; non-neoplastic neurological mouse model indirect/supportive only for BDNF in GBM	Potential relevance to brain tumors through inhibition of tumor growth and induction of apoptosis	Preclinical data; limited direct GBM evidence	[109,117,118]
Quercetin Vegetables and fruits	Modulates Nrf2 and other redox-sensitive pathways through antioxidant and anti-inflammatory activities.	Limited evidence of BDNF regulation in GBM; potential relevance to neurotrophic signaling.	GBM/glioma cell models Direct antiglioma evidence; Speculative BDNF relationship	Reduces IL-6/JAK1/STAT3 signaling, inhibits GBM cell viability, migration, and invasion, and modulates the GSK3β/β-catenin/ZEB1 pathway.	Primarily preclinical data	[104,119]
Vitamin C Fruits and vegetables	Acts as a conventional ROS scavenger and may reduce oxidative damage	Antioxidant-mediated BDNF-associated neuroprotection has been documented in neurological models.	EAE model; observational/meta-analysis of glioma risk Indirect for BDNF; no direct therapeutic GBM evidence	In an observational meta-analysis, higher dietary vitamin C intake was associated with a lower risk of glioma; direct therapeutic effects in GBM remain unknown.	Evidence from neurological and epidemiological models; limited direct GBM evidence	[110,120,121]
Vitamin E Vegetable oils, nuts, and seeds	Reduces oxidative damage through its lipid-soluble antioxidant activity	May preserve BDNF-associated neuroprotective signaling and reduce stress-induced cognitive deterioration.	Non-neoplastic PTSD model Indirect/supportive only	-	Mainly supportive/neuroprotective evidence	[111,120]

Note: BDNF-associated findings and antiglioma activities are summarized as a separate experimental observation and should not be interpreted as evidence of BDNF-dependent antitumor effects unless a direct causal relationship has been experimentally demonstrated. Abbreviations: BDNF—brain-derived neurotrophic factor; Bcl-2—B-cell lymphoma 2; CREB—cAMP response element-binding protein; EGCG—epigallocatechin-3-gallate; ER—endoplasmic reticulum; GBM—glioblastoma; GSK3β—glycogen synthase kinase 3 beta; HT—hydroxytyrosol; IL-6—interleukin-6; JAK1—Janus kinase 1; miRNAs—microRNAs; Nrf2—nuclear factor erythroid 2–related factor 2; p-Akt—phosphorylated protein kinase B; P53—tumor protein p53; PARP—poly(ADP-ribose) polymerase; PTEN—phosphatase and tensin homolog; ROS—reactive oxygen species; STAT3—signal transducer and activator of transcription 3; TMZ—temozolomide; TrkB—tropomyosin receptor kinase B; ZEB1—zinc finger E-box binding homeobox 1.

**Table 2:** Evidence-based evaluation of antioxidant and redox-modulating compounds in glioblastoma: experimental outcomes, clinical studies, and limitations of therapeutic translation.

Antioxidant	In Vitro Glioma/GBM Evidence	Animal Glioma/GBM Evidence	Observational Human Evidence	Early Clinical Evidence	GBM-Specific Clinical Evidence	Reference
Curcumin	Antiproliferative, pro-apoptotic, and TMZ-sensitizing effects; linked with increased ROS and inhibition of Akt/mTOR signaling.	Antitumor and combination therapy effects in experimental GBM settings.	No reliable compound specific GBM survival association established	13 people were enrolled in the presurgical micellar curcuminoid trial, and 10 were finished.	Tumor exposure and viability were shown. No significant OS advantages (14 vs. 13 months; *p* = 0.752) were identified in a phase II analysis of a seven-ingredient mix incorporating curcumin (*n* = 53). Neither study establishes the efficacy of curcumin alone.	[103,105,142].
Resveratrol	Antiproliferative and apoptosis-related effects identified in glioma models.	Reduced tumor burden and antitumor signaling effects in orthotopic rat GBM models.	No reliable resveratrol-specific GBM survival relationship was identified.	Short presurgical resveratrol-copper intervention: ten treated patients and ten untreated controls.	Changes in Ki-67, tumor-associated biomarkers, and gene expression were described; no confirmed OS or PFS improvement, and the results cannot be attributable to resveratrol alone.	[107,143]
EGCG	Modulation of ER stress-related therapeutic response and TMZ sensitivity	Combined EGCG-TMZ treatment increased survival in orthotopic mice GBM models, while EGCG alone did not improve survival.	No qualifying GBM observational survival evidence was identified.	No completed GBM-specific treatment trial was verified.	Preclinical chemosensitization was proven; Clinical efficacy in patients with GBM remains unestablished.	[102]
Hydroxytyrosol	Inhibited viability, migration and stem-like characteristics in T98G and A172 GBM cells	Neuroprotective pathways established outside of GBM; direct animal GBM efficacy not shown by selective evidence.	No qualifying GBM observational survival evidence identified	No completed GBM-specific therapeutic trial verified	Direct in vitro anticancer evidence, but no demonstrated survival benefit or BDNF-mediated therapeutic efficacy	[123,144]
Quercetin	Inhibition of GBM cell growth, migration, and glycolytic metabolism.	Reduced GBM tumor growth and improved survival in experimental animals	There was no evidence of qualifying GBM observational survival found.	There is no verified completed GBM-specific treatment trial.	Animal survival benefit and preclinical anticancer activity; clinical efficacy is unknown	[145]
Vitamin C/pharmacological ascorbate	In GBM models, specific oxidative stress-related cytotoxicity and combination-treatment activity	Antitumor responses investigated in GBM models	Supplements alone do not establish enhanced GBM results.	First-in-human Phase I trial with RT and TMZ, 11 evaluable patients	Feasibility and tolerability are supported; the observed median PFS is 9.4 months and the OS is 18 months. No randomized evidence of superiority in this trial	[124]
Vitamin E	Direct GBM-specific therapeutic evidence insufficient in selected studies	Neuroprotective evidence largely derived from non-GBM models	In a GBM cohort of 470 patients, vitamin E users had a nonsignificant increased mortality estimate (HR 1.54, *p* = 0.09).	No qualifying treatment trial in GBM was validated.	Observational evidence does not show a causal negative or favorable treatment impact.	[146]

## Data Availability

No new data were created or analyzed in this study. Data sharing is not applicable to this article.

## Abbreviations

The following abbreviations are used in this manuscript:

AMPA α	Amino-3-hydroxy-5-methyl-4-isoxazolepropionic acid
ARE	Antioxidant response element
BBB	Blood–brain barrier
BDNF	Brain-derived neurotrophic factor
CREB	cAMP response element-binding protein
CNS	Central nervous system
EGCG	Epigallocatechin-3-gallate
EGFR	Epidermal growth factor receptor
ER	Endoplasmic reticulum
ERK	Extracellular signal-regulated kinase
GBM	Glioblastoma
GPx	Glutathione peroxidase
GSC	Glioma stem cell
GSH	Glutathione
HIF-1α	Hypoxia-inducible factor-1α
HO-1	Heme oxygenase-1
HT	Hydroxytyrosol
IDH	Isocitrate dehydrogenase
IL-6	Interleukin-6
JAK1	Janus kinase 1
Keap1/KEAP1	Kelch-like ECH-associated protein
MAPK	Mitogen-activated protein kinase
MGMT O^6^	methylguanine-DNA methyltransferase
MMP	Matrix metalloproteinase
NF-κB	Nuclear factor kappa
NQO1	NAD(P)H quinone dehydrogenase 1
Nrf2	Nuclear factor erythroid 2-related factor 2
PARP	Poly(ADP-ribose) polymerase
PI3K	Phosphoinositide 3-kinase
PTEN	Phosphatase and tensin homolog
ROS	Reactive oxygen species
SOD	Superoxide dismutase
STAT3	Signal transducer and activator of transcription 3
TMZ	Temozolomide
TrkB	Tropomyosin receptor kinase B
VEGF	Vascular endothelial growth factor
